# Preclinical toxicology and safety pharmacology of the first-in-class GADD45β/MKK7 inhibitor and clinical candidate, DTP3

**DOI:** 10.1016/j.toxrep.2019.04.006

**Published:** 2019-04-19

**Authors:** Laura Tornatore, Daria Capece, Daniel D'Andrea, Federica Begalli, Daniela Verzella, Jason Bennett, Gary Acton, Elizabeth A. Campbell, James Kelly, Michael Tarbit, Nigel Adams, Selina Bannoo, Antonio Leonardi, Annamaria Sandomenico, Domenico Raimondo, Menotti Ruvo, Angela Chambery, Metod Oblak, Magda J. Al-Obaidi, Richard S. Kaczmarski, Ian Gabriel, Heather E. Oakervee, Martin F. Kaiser, Ashutosh Wechalekar, Reuben Benjamin, Jane F. Apperley, Holger W. Auner, Guido Franzoso

**Affiliations:** a CCSI, Department of Medicine, Imperial College London, London, UK; b Cancer Research UK Centre for Drug Development, London, UK; c C&C Management Consulting Ltd, Exmouth, UK; d Alpha Preclinical Consultancy, Halifax, UK; e Independent Consultant, Royston, UK; f In2Phase Ltd, Welwyn Garden City, UK; g Department of Molecular Medicine, University of Naples Federico II, Naples, Italy; h IBB-CNR and CIRPeB, "Federico II" University of Naples, Naples, Italy; i Department of Molecular Medicine, Sapienza University of Rome, Rome, Italy; j DiSTABiF, University of Campania "Luigi Vanvitelli", Caserta, Italy; k West Middlesex University Hospital, Isleworth, Greater London, UK; l Haematology Department, Chelsea and Westminster Hospital, London, UK; m London Haematology Limited, London, UK; n Barts Cancer Centre, St Bartholomew's Hospital London, London, UK; o Division of Molecular Pathology, The Institute of Cancer Research, London, UK; p Royal Free London NHS Foundation Trust, London, UK; q Department of Haematology, King's College Hospital, London, UK; r Centre for Haematology, Imperial College, London, UK; s Cancer Cell Protein Metabolism, Department of Medicine, Imperial College London, London, UK

**Keywords:** NF-κB, GADD45β, Multiple myeloma, Cancer, Pharmacology

## Abstract

Aberrant NF-κB activity drives oncogenesis and cell survival in multiple myeloma (MM) and many other cancers. However, despite an aggressive effort by the pharmaceutical industry over the past 30 years, no specific IκBα kinase (IKK)β/NF-κB inhibitor has been clinically approved, due to the multiple dose-limiting toxicities of conventional NF-κB-targeting drugs. To overcome this barrier to therapeutic NF-κB inhibition, we developed the first-in-class growth arrest and DNA-damage-inducible (GADD45)β/mitogen-activated protein kinase kinase (MKK)7 inhibitor, DTP3, which targets an essential, cancer-selective cell-survival module downstream of the NF-κB pathway. As a result, DTP3 specifically kills MM cells, *ex vivo* and *in vivo*, ablating MM xenografts in mice, with no apparent adverse effects, nor evident toxicity to healthy cells. Here, we report the results from the preclinical regulatory pharmacodynamic (PD), safety pharmacology, pharmacokinetic (PK), and toxicology programmes of DTP3, leading to the approval for clinical trials in oncology. These results demonstrate that DTP3 combines on-target-selective pharmacology, therapeutic anticancer efficacy, favourable drug-like properties, long plasma half-life and good bioavailability, with no target-organs of toxicity and no adverse effects preclusive of its clinical development in oncology, upon daily repeat-dose administration in both rodent and non-rodent species. Our study underscores the clinical potential of DTP3 as a conceptually novel candidate therapeutic selectively blocking NF-κB survival signalling in MM and potentially other NF-κB-driven cancers.

## Highlights

• DTP3 eliminates any viable MM cells in mice upon i.v. bolus administration.
• DTP3 exhibits highly favourable PK and ADME profiles, with long plasma half life.
• DTP3 had no adverse effect on vital organ systems in GLP safety pharmacology studies.
• DTP3 was tolerated in repeat-dose 28-day toxicity studies with wide exposure margins.

## Graphical abstract

**Figure:**
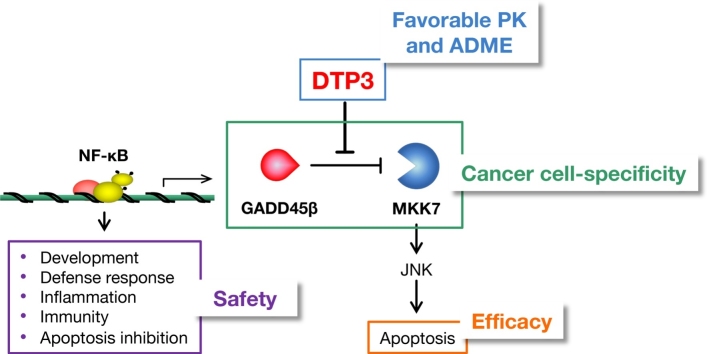

## 1 Introduction

NF-κB transcription factors are aberrantly activated in most human cancers, including multiple myeloma (MM), where they drive oncogenesis, therapy resistance and malignant cell survival largely by upregulating genes that suppress cancer-cell apoptosis. As such, the NF-κB pathway represents an attractive therapeutic target in oncology [[1], [2], [3], [4]]. However, while with an increased understanding of cancer core vulnerabilities, there has been a corresponding development of effective targeted therapeutics, leading to improved clinical outcomes in oncology, pharmacologically targeting the NF-κB pathway has proven an insurmountable challenge. This dismal outcome of the 30-year search for a clinically useful NF-κB inhibitor is epitomised by the ill-fated pursuit of agents targeting IKKβ, the molecular gatekeeper of the cellular pathways for NF-κB activation, which, following its discovery in 1997, had re-focused the drug-discovery effort to therapeutically block NF-κB [2,5,7]. However, this effort came to an abrupt end 10 years later, when IKKβ-targeting agents were found to cause severe systemic inflammation and animal lethality in mouse models [7]. Further studies confirmed this paradoxical pro-inflammatory effect of IKKβ inhibitors in human trials, while exposing a series of additional on-target dose-limiting toxicities, including hepatotoxicity and numerous immunodeficiencies, resulting from the general suppression of the NF-κB pathway [2,8,9].

The barrier to effectively targeting NF-κB has been to achieve safe NF-κB inhibition, which requires preserving the pleiotropic and ubiquitous physiological functions of the NF-κB pathway [2,6,10]. Since constitutive NF-κB activity drives oncogenesis by upregulating antiapoptotic genes [2,10], we sought to overcome this barrier by targeting the non-redundant, cancer-specific downstream effectors of the oncogenic NF-κB survival function, rather than NF-κB itself [11]. We tested this principle in MM, an incurable plasma-cell malignancy and the paradigm for NF-κB-driven cancers [12]. Virtually all MM cases display constitutive NF-κB signalling, an addiction to NF-κB activity for survival, and susceptibility to apoptosis upon IKKβ/NF-κB-pathway inhibition [1]. As such, MM provided an ideal testing ground for our novel therapeutic approach.

The current treatments for MM include chemotherapy, steroids, immunomodulatory (IMiD) agents (*i.e.* thalidomide, lenalidomide and pomalidomide), and proteasome inhibitors (*i.e.* bortezomib and carfilzomib), with newer agents in development including anti-CD38 antibody and CAR-T immunotherapy, and autologous stem-cell transplantation being indicated in selected patients. However, none of these treatments generally achieves lasting remissions, leading to relapse and/or refractory disease [[12], [13], [14], [15]]. Moreover, while IMiDs and proteasome inhibitors have been shown to impact NF-κB signalling, these agents have broad biological activities, lack specificity for the NF-κB pathway, and, importantly, afford their clinical effects in MM *via* NF-κB-independent mechanisms [1,16,17]. Consequently, there is an urgent need for an entirely new approach to safely block aberrant NF-κB signalling in MM and other NF-κB-driven cancers.

Recently, we identified the complex formed by NF-κB-regulated antiapoptotic factor, GADD45β, and the c-Jun N-terminal kinase (JNK)-activating kinase, MKK7, as an essential, cancer-selective survival module downstream of NF-κB and novel therapeutic target in MM [11,[18], [19], [20]]. We showed that elevated *GADD45B* expression in MM cells correlates with poor clinical outcome and promotes malignant cell survival by suppressing JNK-driven apoptosis through a mechanism that depends upon the GADD45β-mediated binding to and inhibition of MKK7 [11,21]. Crucially, most normal cells do not require GADD45β for their survival [22], and, unlike mice lacking the NF-κB/RelA subunit or any core components of the IKK complex, *Gadd45b* knock-out mice are viable, fertile and die of old age [2,23,24]. Therefore, to selectively block oncogenic NF-κB signalling in MM, we therapeutically targeted the downstream GADD45β/MKK7 survival complex. Accordingly, we developed a D-tripeptide inhibitor of this complex, DTP3, which specifically binds to MKK7 *via* a mechanism that effectively disrupts the GADD45β/MKK7 interaction [11,21]. As a result of this therapeutic mode of action, DTP3 specifically kills MM cells exhibiting elevated GADD454β expression, *ex vivo* and *in vivo*, by inducing MKK7/JNK-driven apoptosis, and, importantly, does not appear to be toxic to normal tissues. Strikingly, upon systemic administration, DTP3 caused a complete regression of established subcutaneous MM xenografts in mice, while extending animal survival in orthotopic MM models, with good tolerability and no apparent adverse effects. Due to its mode of action, operating downstream of NF-κB, DTP3 also exhibits the capacity to synergise with bortezomib and bypass drug resistance to conventional anti-MM therapies, including steroids, IMiDs and proteasome inhibitors.

Therefore, to translate these advantageous pharmacological properties into healthcare benefit, we sought to initiate clinical trials of DTP3 in patients with relapsed or refractory MM. In this study, we report the results from the preclinical safety pharmacology, toxicology, PK and PD studies of DTP3, leading to the regulatory approval for clinical trials of this novel anticancer candidate therapeutic in patients with relapsed or refractory MM. Our results demonstrate that DTP3 displays on-target-selective pharmacology, no significant adverse effects against vital organ systems, and favourable PK and absorption, distribution, metabolism and excretion (ADME) profiles, while retaining its therapeutic efficacy following intravenous bolus administration. DTP3 was tolerated upon daily administration over a 28-day period in both rodent and non-rodent species, demonstrating no target-organs of toxicity, no adverse effect precluding its clinical development in oncology, and a wide preclinical therapeutic window. As such, DTP3 introduces into oncology a novel therapeutic mode of action selectively blocking pathogenic NF-κB survival signalling in cancer cells, while overcoming the dose-limiting toxicities of conventional IKKβ/NF-κB-targeting drugs. Indeed, due its cancer-selective pharmacology and capacity to bypass drug resistance and synergise with bortezomib, DTP3 represents a significant clinical opportunity, which could translate into a safe and effective anticancer therapeutic to treat patients with relapsed/refractory MM and potentially other recalcitrant NF-κB-driven cancers.

## 2 Results

### 2.1 Secondary pharmacology and drug-drug interactions

**Fig. 1:**
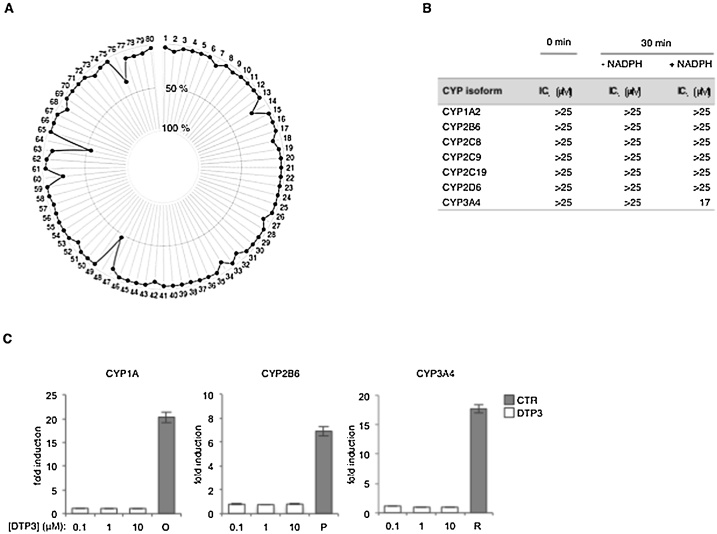
The secondary pharmacology and drug-drug interaction potential of DTP3. **A**, The profile of DTP3 in radio-ligand competition binding assays against a panel of 80 validated drug targets (see also Supplementary Fig. 1A). Values represent the mean percentage of inhibition of the binding of target-specific control ligands in the presence of DTP3 (10 μM) relative to the binding observed in the absence of DTP3 (n = 2). Inhibition greater than 50% was considered to represent a significant effect for the purpose of conducting further investigations. 48, μ-opioid peptide (MOP) receptor; 64, Sigma receptors (non-selective). B, Time-dependent inhibition assays showing the DTP3-mediated inhibition of the indicated cytochrome P450 (CYP) isoforms in the absence (0 min) or presence of a 30-minute pre-incubation (30 min) of human liver microsomes with or without NADPH, as shown. IC_50_ values denote the concentrations of DTP3 resulting in a 50% inhibition of the formation of CYP isoform-specific metabolites. **C**, Enzymatic assays showing the fold induction of the indicated CYP isoforms following a 72-hour treatment of human hepatocytes with either DTP3, at the indicated concentrations, or isoform-specific control inducers, relative to vehicle control. Values denote the mean fold inductions ± SD (n = 3) of isoform-specific metabolite levels following hepatocyte treatment. O, omeprazole; P, phenobarbital; R, rifampicin.

We previously reported that DTP3 displayed no significant off-target effects in a panel of 142 human kinases, and that its therapeutic activity in sensitive MM cell lines was completely abrogated by the RNA interference-mediated inhibition of MKK7, its pharmacological target, suggesting it exhibits high target specificity [11]. To further evaluate the potential of DTP3 for mediating secondary pharmacological effects, ahead of the regulatory submissions, we used a conventional *in-vitro* selectivity screen. Except for a weak interaction with Sigma (non-selective) and μ-opioid peptide (MOP) receptors, DTP3 demonstrated no significant off-target effect when profiled in radioligand competition binding assays against a panel of 80 validated drug targets, including enzymes, receptors, and transporters (Fig. 1A, Supplementary Fig. 1A). On further investigation, DTP3 displayed a low binding affinity for Sigma receptors, with an IC_50_ value of 13 μM and inhibition constant (K_i_) of 10 μM (Supplementary Fig. 1B-C). At higher concentrations (*i.e.*, 100 μM), DTP3 also exhibited a weak antagonist effect on Sigma (Supplementary Fig. 1D). While these weak *in-vitro* interactions of DTP3 with Sigma and MOP receptors identify a potential for secondary pharmacological activities, these effects were observed at relatively high drug concentrations, significantly higher than the therapeutically effective *in-vivo* plasma concentrations [11], and moreover, are not relevant, at least from a regulatory perspective, for a clinical drug candidate in oncology.

To further understand the pharmacology of DTP3, we examined the potential of this agent for mediating drug-drug interactions *via* drug-metabolising enzymes of the cytochrome P450 (CYP) family. Upon evaluation in human liver microsomes, *ex vivo*, DTP3 demonstrated a weak time-dependent inhibitory effect on CYP3 A4, but no reversible inhibition of any other major human CYP isoform, as shown by its relatively high IC_50_ values in the presence or absence of NADPH. (Fig. 1B, Supplementary Fig. 1E-F). Moreover, DTP3 did not induce any major human CYP isoform in human hepatocytes, *ex vivo* (Fig. 1C). Given the weak NADPH-dependent inhibitory effect of DTP3 on CYP3 A4 and the high frequency of drug-mediated effects on this CYP isoform by the medicines in current clinical use, this potential for engaging in drug-drug interactions *via* CYP3 A4 poses no problem to the clinical development of DTP3 in oncological patients. Collectively, our findings underscore the overall limited potential of DTP3 for mediating preclusive off-target effects and some potential for mediating weak drug interactions *via* CYP3 A4.

### 2.2 Non-specific cytotoxicity and genotoxicity

**Fig. 2:**
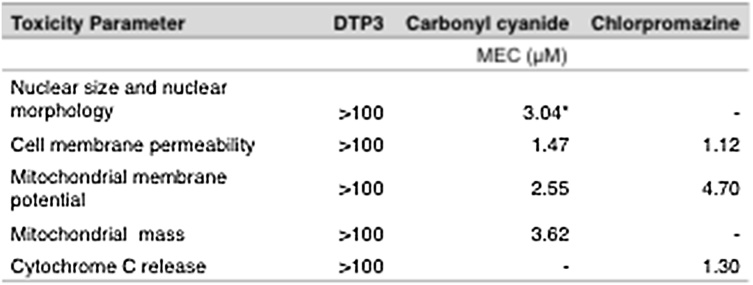
Absence of off-target hepatocellular cytotoxicity by DTP3. Table reporting the results of cytotoxicity assays performed in HepG2 cells following treatment with DTP3 or positive control compounds (*i.e.*, carbonyl cyanide and chlorpromazine), as indicated, at the concentrations of 0.04, 0.1, 0.4, 1.0, 4.0, 10, 40 and 100 μM. Reported is the minimum effective concentration (MEC) of each compound resulting in a significant cytotoxic effect relative to vehicle control. Values denote means. -, not applicable; *, non-statistically significant fit.

We investigated the non-specific cytotoxic potential of DTP3 in the human hepatocellular carcinoma cell line, HepG2, which does not express GADD45β (Supplementary Fig. 2A), using a multi-parametric toxicity assay, *in vitro*. As expected, upon evaluation at concentrations up to 100 μM, DTP3 induced no change in any of the cytotoxicity parameters investigated, including cytochrome *c* release (Fig. 2), thus excluding any non-specific drug-dependent cytotoxic effect in this experimental system. To investigate the mutagenic potential of DTP3, we conducted a Good Laboratory Practice (GLP)-compliant bacterial reverse mutation study, using five different histidine-requiring strains of *S. typhimurium*, each reporting on distinct classes of genetically active compounds. As shown in Supplementary Fig. 2B-C, whereas control agents caused a significant increase in the number of revertant colonies with each of the tester strains evaluated, the exposure to DTP3 resulted in no change in colony numbers, compared to vehicle control, irrespective of the presence or absence of metabolic activation by Aroclor 1254-induced rat liver post-mitochondrial S9 fraction. Hence, DTP3 exhibited no mutagenic activity in this assay.

### 2.3 The safety pharmacology of DTP3

**Fig. 3:**
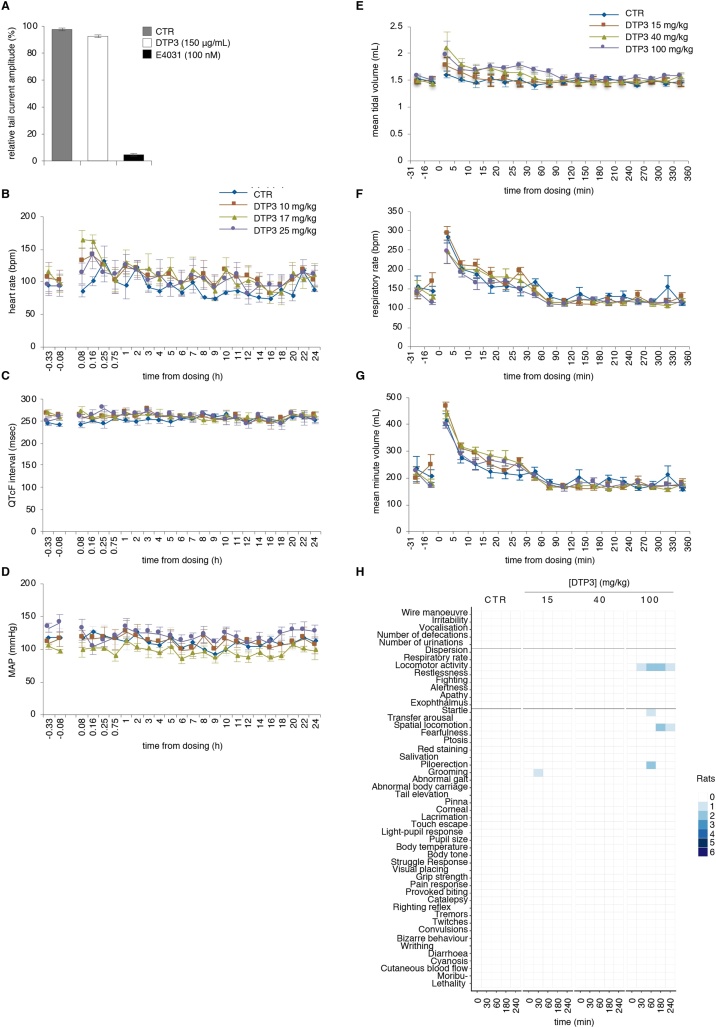
The safety pharmacology of DTP3. A, hERG assays showing the percentage of inhibition of hERG-dependent outward tail currents in HEK-293 cells following treatment with vehicle (CTR), DTP3 or the selective *I_Kr_* blocker, E-4031, at the indicated concentrations. Values represent the mean percentages ± SD (n = 3) of the recorded current inhibition relative to pre-treatment voltage-clamp recordings from individual HEK-293 cells. B-D, Radio-telemetry recordings of the indicated cardiovascular parameters at the time shown in conscious, freely moving dogs following rapid intravenous infusion of vehicle control or the reported doses of DTP3. B, bpm, beats per minute. C, QTc interval, corrected QT interval. D, MAP, mean arterial blood pressure. E-G, Whole-body plethysmography recordings of the indicated respiratory parameters at the time shown in conscious, freely moving rats following rapid intravenous infusion of vehicle control or the reported doses of DTP3. B-G, Values denote means ± SD; n = 4 (B-D), n = 6 (E-G). H, Heat-map showing the number of rats exhibiting post-dosing abnormalities in autonomic or locomotor activity and/or general behaviour (n = 6), according to Irwin's method, at the times shown after treatment with vehicle control or the indicated doses of DTP3 as in (E-G). A-H, assays were conducted in compliance with GLP.

We performed a GLP-compliant human ether-à-go-go related gene (hERG) study, *in vitro*, to assess the potential of DTP3 for causing QT-interval prolongation. As shown in Fig. 3A, DTP3 produced no inhibition of the potassium ion channel, hERG, when used at the concentration of 150 μg/mL. By contrast, exposure to the selective rapid delayed rectifier current (*I_Kr_*) blocker, E-4031, resulted in a complete suppression of hERG-mediated tail currents. These results demonstrate the low potential of DTP3 for causing a long-QT syndrome, a common cause of life-threatening cardiac arrhythmias.

Next, we conducted a safety risk assessment of DTP3 on vital organ systems by performing GLP-compliant safety pharmacology studies, *in vivo*, following short infusion (over 10 min) by the intravenous route of administration, as the clinical therapeutic route of choice. Consistent with the results from the hERG study (Fig. 3A), intravenous DTP3 administration at doses up to 25 mg/kg produced no clinically significant effect on any of the cardiovascular or haemodynamic parameter evaluated in the conscious, freely moving dog, including ECG waveform, QT_C_ interval, mean arterial pressure (MAP), and heart rate (Fig. 3B-D). A transient increase in heart rate was noted in some animals at the first 2 time points, within 10 min of dosing, but this increase was not dose-dependent and therefore considered not to be drug related. DTP3 was similarly assessed for potential adverse effects on respiratory function in the freely moving conscious rat. As shown in Fig. 3E–G, upon rapid DTP3 intravenous infusion at the dose levels of 15, 40 or 100 mg/kg, drug-treated animals demonstrated no significant change in any respiratory parameter, including tidal volume, respiration rate and minute volume, as compared to the vehicle-treated group. The same dose levels and regimen of administration were used in the rat to also evaluate the effects of DTP3 on the CNS, according to Irwin’s method. DTP3 induced no change in body temperature, nor general behaviour, nor autonomic nor motor activity at the dose level of either 15 or 40 mg/kg (Fig. 3H). A mild and transient increase in locomotor activity was observed in a minority of the animals treated at the dose level of 100 mg/kg (Fig. 3H), but this effect was considered not to be drug-related, as it was not observed in the 28-day repeat-dose toxicity study, and DTP3 was found not to cross the blood-brain barrier to any significant extent (discussed below). Furthermore, such potential effect on CNS function would be of no relevance from a regulatory perspective for the safety assessment of an oncological drug. No further changes in body temperature, nor behaviour were observed following DTP3 administration at 100 mg/kg (Fig. 3H). We concluded from these GLP-compliant safety pharmacology studies that DTP3 does not significantly adversely affect the CNS, nor the cardiovascular nor respiratory system, at any of the dose levels investigated. Collectively, these results underpin the excellent safety pharmacology of DTP3 and support the progression of this novel candidate therapeutic into clinical evaluation in oncology.

### 2.4 The PK profile of DTP3

A series of PK and toxicokinetic (TK) studies were conducted in mouse, rat and dog. The dose-related PK values derived from these studies demonstrated that, following intravenous administration of DTP3, maximum plasma concentrations (C_max_) and systemic exposures (AUC_0-t_) increased in a linear manner with dose level in each species, indicating that there was no saturation of clearance at the higher dose levels (Supplementary Fig. 3A-C). There were no gender differences in PK parameters (Supplementary Fig. 3A–B). As expected, the mean C_max_ values of DTP3 coincided with the end of the injection or infusion. Thereafter, the plasma concentrations of DTP3 declined in a biphasic manner in both rodent and non-rodent species, demonstrating a marked distribution phase followed by a longer terminal elimination phase.

**Fig. 4:**
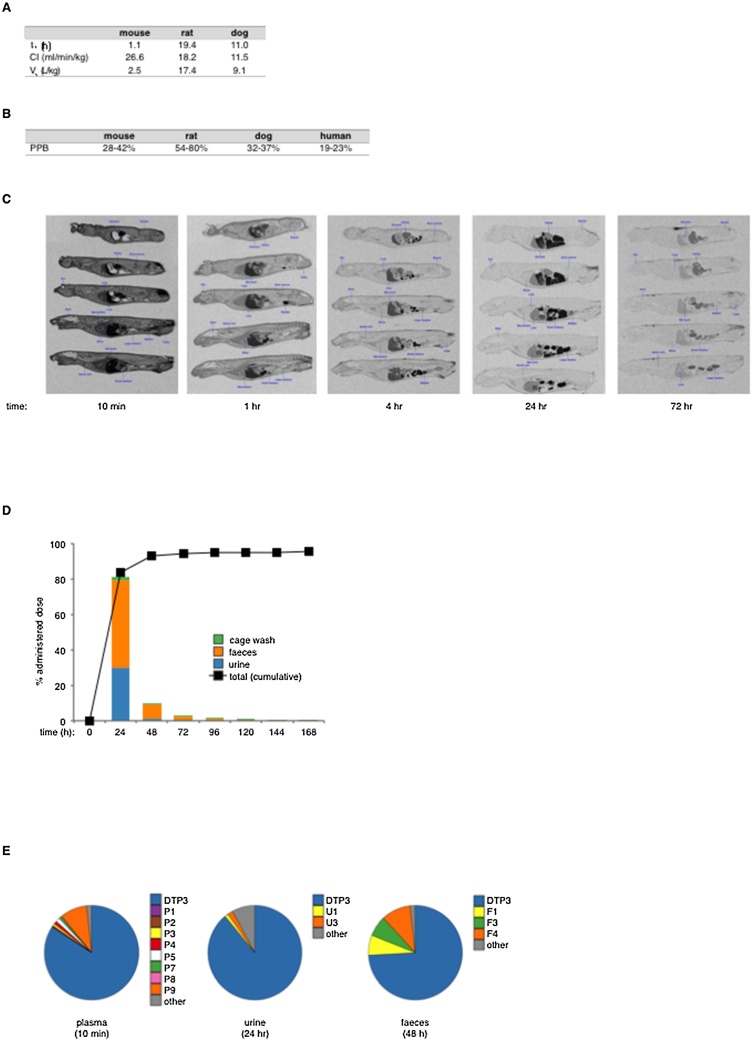
The PK and ADME profiles of DTP3. A, Table reporting the mean values of the main PK parameters of DTP3 in mouse, rat and dog following intravenous administration by rapid infusion (rat and dog) or injection (mouse). t_½_, terminal half-life; CL, clearance; V_a_, volume of distribution area. B, *In-vitro* plasma protein binding (PPB) values of DTP3 in mouse, rat, dog and man, as shown. Values denote the percentages of bound drug fraction. C, Representative quantitative whole-body autoradiographs (QWBA) showing the tissue distribution of DTP3-related radioactivity in rat at the times indicated, following rapid intravenous infusion of 15 mg/kg of [^14^C]-labelled DTP3. D, Liquid scintillation counting (LSC) showing the relative excretion of DTP3-related radioactivity in urine, faeces and cage washes in rats (columns) at the times indicated following administration of [^14^C]-labelled DTP3 as in (C). Also shown are the mean cumulative excretion values of DTP3-related radioactivity (line). Values denote the mean percentages of total administrated radioactivity (n = 3). E, High-resolution LC–MS/MS analysis showing the mean percentages of the indicated radioactive metabolites in chromatographic profiles from plasma, urine and faeces at the times shown following administration of [^14^C]-labelled DTP3 as in (**C**). Compounds are identified by the following matrix codes: P, plasma (n = 6); U, urine (n = 3); F, faeces, (n = 3). Metabolites assigned by LC–MS/MS are as follows: P9, U3 and F4, product of the oxidative deamination of DTP3; P7 and F3, methoxy-DTP3.

Based on these data, the overall mean volume of distribution (V_a_) values were approximately 2 L/kg in mouse, 17 L/kg in rat, and 9 L/kg in dog, indicating that DTP3 largely distributed into tissues across species (Fig. 4A). The mean plasma clearance (CL) of DTP3 was moderate in all species, exhibiting values of approximately 27 mL/min/kg, 18 mL/min/kg and 11 mL/min/kg in mouse, rat and dog, respectively, corresponding in each case to approximately 30% of liver blood flow (Fig. 4A). These values are in keeping with a slow rate of elimination of DTP3, resulting in the relatively long overall mean terminal half-lives (t_½_) of 19 and 11 h in rat and dog, respectively (Fig. 4A). T_½_ values appeared to be instead considerably shorter in mouse, ranging between 1 and 1.5 h. Upon repeated rapid daily infusion, the corresponding mean C_max_ and AUC_0-t_ values were slightly higher on day 28 than day 1 in both rat and dog, reflecting the long terminal plasma half-lives of DTP3 in these species and, again, were essentially proportional with respect to dose level, indicating that there was no marked accumulation of DTP3, nor any alteration in systemic clearance, with increased dose or repeated administration (Supplementary Fig. 3A–B).

From these preclinical data, we sought to project a human plasma half-life, using a conventional scaling methodology based on inter-species differences in liver blood flow, while assuming that systemic clearance would be the same proportion of liver blood flow in all species, and that volume of distribution would also be the same [25,26]. The resulting projections for human plasma half-life were 33 and 18 h on the basis of the PK profiles observed in rat and dog, respectively, suggesting the suitability of DTP3 for a clinically advantageous, long-term intravenous dosing regimen. Interestingly, at least in the mouse, the mean systemic exposures and elimination half-lives of DTP3 were broadly equivalent within a wide dosing range, following either subcutaneous or intravenous injection (Supplementary Fig. 3C-D), suggesting a good relative systemic bioavailability of DTP3 by the subcutaneous administration route, which could provide a valuable adjunct to the clinical use of this novel agent.

### 2.5 The ADME profile of DTP3

The *in vitro* plasma protein binding (PPB) of DTP3 was consistently low across species, with percentage values of bound drug fraction of around 20–40% in mouse, dog and man, and somewhat higher values and a degree of variability between studies in rat (Fig. 4B). The tissue distribution of DTP3 was investigated upon intravenous administration of [^14^C]-labelled drug material to the rat. As shown in Fig. 4C, DTP3 rapidly and extensively distributed to most tissues. The highest levels of radioactivity were recorded in urine, liver, kidney cortex, bile ducts and urinary bladder wall (Supplementary Fig. 3E), denoting the main routes of elimination (discussed below). Notably, no DTP3-related radioactivity was detected in brain, nor spinal cord, indicating that DTP3 did not cross the blood-brain barrier to a significant extent (Fig. 4C, Supplementary Fig. 3E).

Approximately 90% of the total administered radioactivity was excreted in urine and faeces within 48 h of dosing (Fig. 4D). Renal elimination was relatively low, comprising approximately 30% of the total administered dose. Faecal elimination represented the predominant route of excretion, with approximately 60% of radioactivity being eliminated by this route (Fig. 4D), suggesting some biliary involvement. The remaining radioactivity was largely recovered in cage washings, and no more than 0.5% of the total administered drug material was detected in carcasses at the end of the sampling period, indicating that DTP3 had not accumulated in tissues.

Upon evaluation of its stability in hepatocytes from rat, dog, monkey and man, *ex vivo*, DTP3 displayed no significant metabolism in any of these species (Supplementary Fig. 3F), suggesting that it is a low-clearance compound, an advantageous drug-like characteristic. In keeping with these *ex-vivo* results, no major drug metabolites were detected in either plasma, urine or faeces, following intravenous administration of DTP3 to the rat (Fig. 4E). In plasma, DTP3 was by far the most abundant radiolabelled chemical species (Fig. 4E; see also Supplementary Fig. 3G). The LC–MS/MS analysis of plasma demonstrated the presence of up to eight DTP3 metabolites, but collectively these amounted to no more than 15% of the total radioactivity (Fig. 4E, Supplementary Fig. 3G). Similarly, DTP3 accounted for 89% and 74% of the total radioactivity detected in urine and faeces, respectively (Fig. 4E). We concluded that DTP3 is not significantly metabolically cleared, *in vivo*. Collectively, our findings demonstrate the favourable PK and ADME profiles of DTP3 and reinforce the clinical potential of this novel candidate anticancer therapeutic.

### 2.6 The therapeutic efficacy of DTP3 upon intravenous bolus injection

**Fig. 5:**
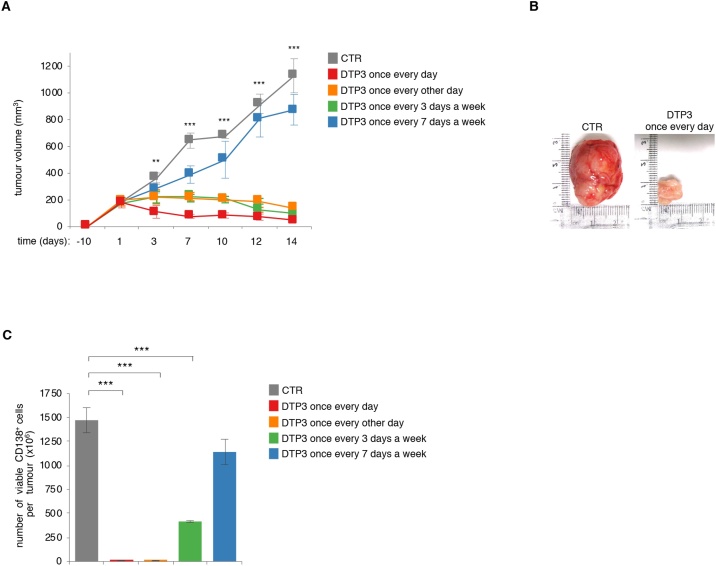
The therapeutic efficacy of DTP3 in a mouse MM xenograft model upon intravenous bolus injection by a clinically suitable dosing schedule. A, Volumes of subcutaneous U266 MM xenografts in mice at the time shown following treatment with vehicle control (CTR) or 10 mg/kg of DTP3, according to the indicated intravenous dosing schedule. Statistical comparisons are shown between the vehicle-treated group and the group treated with DTP3 once every day. B, Images of representative tumours from the indicated treatment groups in (A) at day 14. C, Trypan blue exclusion assays showing the number of live MM (CD138^+^) cells in tumours from the groups in (A) at the experimental endpoint on day 14. **A** and **C**, Values denote means ± SEM (vehicle-treated group, n = 6; DTP3-treated groups, n = 3). **, p < 0.01; ***, p < 0.001.

We previously showed that DTP3 was effective in eliminating MM xenografts in both subcutaneous and orthotopic mouse models, upon administration by continual infusion [11]. However, since this administration regimen is impractical for clinical use and development, we sought to determine whether DTP3 retained its therapeutic efficacy following administration by intravenous bolus injection, using a previously characterised subcutaneous MM xenograft model and different potential clinical dosing schedules. As shown in Fig. 5A–B, at the experimental end-point, all mice in the vehicle-treated group had developed large tumours, whereas mice treated with 10 mg/kg of DTP3 once every day, once every other day, or once every 3 days demonstrated a dramatic tumour shrinkage. By contrast, DTP3 administration once a week produced no significant effect on tumour volume (Fig. 5A). Interestingly, an analysis of residual MM cells in mice established that DTP3 achieved a virtually complete elimination of any viable CD138^+^ cells when administered once every day or once every other day, whereas DTP3 administration once every 3 days was significantly less effective in killing MM cells (Fig. 5C). These data demonstrate the potent therapeutic efficacy of DTP3 upon administration by a clinically advantageous intravenous bolus injection schedule and establish the optimal clinical dosing frequency of approximately once every other day for the first-in-human trial of DTP3 in MM patients.

To verify the therapeutic mode of action of DTP3, following administration by the proposed clinical intravenous dosing schedule, we evaluated the induction of JNK signalling and apoptosis in tumour cells from subcutaneous MM xenografts. As shown in Supplementary Fig. 4A–B, DTP3 administration by this dosing schedule induced a strong pharmacodynamic response, denoting therapeutic target engagement, in virtually all CD138^+^ cells within tumours, as shown by the near-complete shift in the signals reporting JNK phosphorylation and caspase-3 cleavage, and the marked increase in the percentage of MM cells exhibiting a sub-G_1_ DNA content, a hallmark of apoptosis. By contrast, DTP3 administration had no effect on ERK phosphorylation in MM cells, confirming the specificity of its therapeutic mode of action, *in vivo* (Supplementary Fig. 4A). These results confirm the DTP3 mode of action, *in vivo*, and demonstrate the suitability of the FACS-based methodology for detecting a cancer-selective pharmacodynamic response in MM patients from the first-in-human study of DTP3.

### 2.7 The toxicological profile of DTP3

Dose-ranging studies were conducted in rodent and non-rodent species to determine the maximum tolerated dose (MTD) of DTP3 in these species. In the rat, DTP3 administration resulted in the death of both animals treated at the dose level of 150 mg/kg. Two out of four animals treated with 125 mg/kg of DTP3, as well as two controls, also died (data not shown). All deaths occurred during or immediately after dosing, and there were no drug-related clinical or pathological findings in any of the decedents. Conversely, DTP3 was tolerated at dose levels up to 100 mg/kg, causing only mild and transient clinical signs and no clinically significant pathological changes, nor any changes in body weight, haematology or clinical chemistry parameters. Accordingly, the dose level of 100 mg/kg was declared the MTD in the rat.

**Fig. 6:**
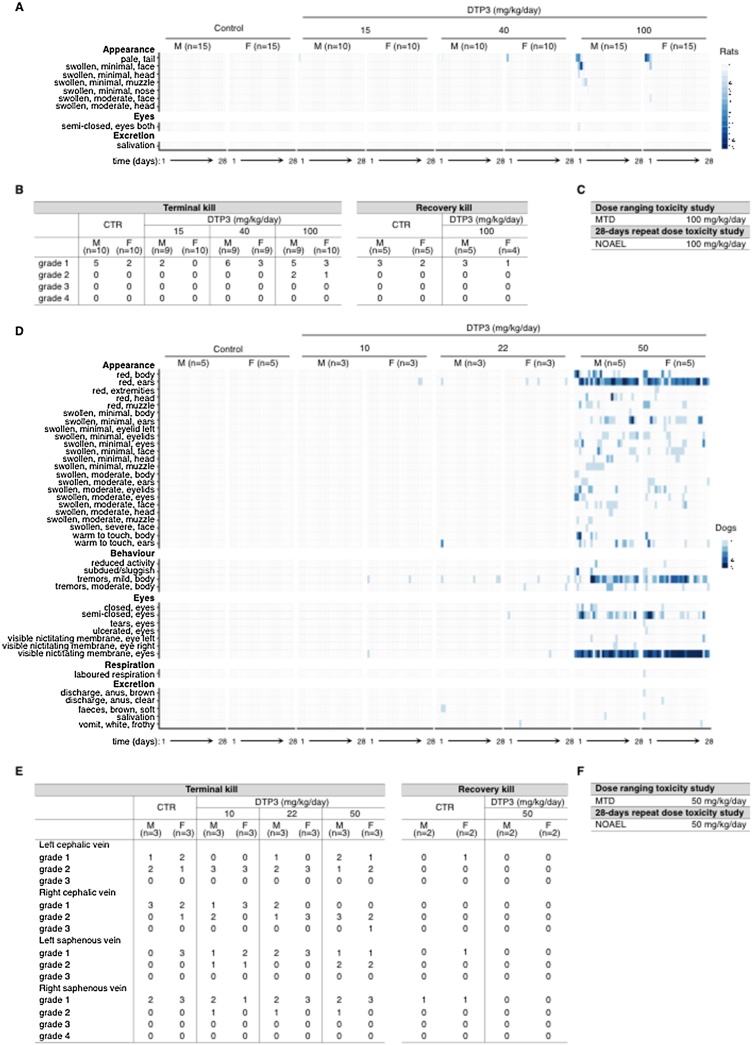
The toxicological profile of DTP3. A, Heat-map showing the number of rats manifesting the indicated post-dosing observations at the times shown following daily rapid intravenous infusion of vehicle control or the specified doses of DTP3 during the 28-day repeat-dose toxicity study (please refer to Supplementary Fig. 5B for the list of in-life procedures undertaken). B, Table reporting the number of rats exhibiting kidney tubular basophilia at microscopic observation at the terminal or recovery kill, as shown, following treatment with vehicle control or DTP3 as in (A). Also reported is the severity of the microscopic renal findings (please refer to Supplementary Fig. 5D-E for the list of post-mortem procedures undertaken). C, Table reporting the maximum tolerated dose (MTD) and no observed adverse effect level (NOAEL) of DTP3 in the rat. D, Heat-map showing the number of dogs manifesting the indicated post-dosing observations at the times shown following daily rapid intravenous infusion of vehicle control or the specified doses of DTP3 during the 28-day repeat-dose toxicity study (please refer to Supplementary Fig. 5B for the list of the in-life procedures undertaken). E, Table reporting the number of dogs exhibiting fasciitis/fibrosis at the indicated injection sites at microscopic observation at the terminal or recovery kill, as shown, following treatment with vehicle control or DTP3 as in (D). Also reported is the severity of the microscopic finding (please refer to Supplementary Fig. 5G for the list of post-mortem procedures undertaken). F, Table reporting the MTD and NOAEL of DTP3 in the dog. A–B and D-E, M, male; F, female. B and E, Grade 1, minimal; grade 2, slight; grade 3, moderate; grade 4, severe.

The GLP-compliant 28-day repeat-dose toxicity study was therefore conducted upon daily rapid intravenous infusion of DTP3 in the rat at the dose levels of 15, 40 and 100 mg/kg/day (Supplementary Fig. 5A). Delayed-onset toxicity and reversibility of toxicity were assessed following an additional 28-day treatment-free period (Supplementary Fig. 5A). The vast majority of the over one hundred animals given DTP3 completed the 28-day treatment period with no or only few minor adverse effects. Post-dosing observations were limited to minor and transient clinical signs, such as swollen face, head and/or muzzle and semi-closed eyes, only noted in the animals receiving 100 mg/kg/day of DTP3 (Fig. 6A). Clinical signs were invariably observed immediately after dosing and generally resolved within 1 h and in all cases had disappeared by the end of the day. There were no ophthalmic findings, nor any treatment-related changes on body weight nor food consumption, nor were there any effects on haematology, clinical chemistry or urinalysis parameters which were considered to be drug-related or toxicologically significant (Supplementary Fig. 5B-C; see list of in-life procedures).

At the terminal and recovery kills, there were no treatment-related changes in organ weights, nor any macroscopic findings suggestive of either a local or systemic effect of DTP3 (Supplementary Fig. 5D). Microscopic findings were confined to a minor increase in the incidence and severity of kidney tubular basophilia, noted in a few rats treated at the dose level of 100 mg/kg/day (Fig. 6B; see also Supplementary Fig. 5D-E). In the absence of any other findings or any urinalysis or clinical pathology correlates (Supplementary Fig. 5C), this minor histological change was considered not to be adverse nor, consequently, dose limiting. At the recovery kill, there was a complete reversal of the microscopic renal findings observed at the terminal kill (Fig. 6B, Supplementary Fig. 5E). We concluded that, following daily infusion over a 28-day period in the rat, DTP3 was tolerated at dose levels up to 100 mg/kg/day. Accordingly, the No Observed Adverse Effect Level (NOAEL) in this species was considered to be the maximum administered dose of 100 mg/kg/day (Fig. 6C).

During the dose-ranging study in the dog, there were no decedents. In the only animal treated at the dose level of 75 mg/kg, DTP3 administration produced vomiting, which was considered to be dose limiting (data not shown). Conversely, DTP3 was tolerated at dose levels up to 50 mg/kg/day, causing only mild and transient clinical signs, and no drug-related changes in bodyweight, nor any clinical pathology, nor macroscopic nor microscopic parameters. Based on these results, the dose level of 50 mg/kg was declared the MTD in the dog.

Accordingly, the GLP-compliant 28-day repeat-dose toxicity study in this species was conducted upon daily rapid intravenous infusion of DTP3 at the dose levels of 10, 22 or 50 mg/kg/day, and delayed-onset toxicity and reversibility of toxicity were subsequently assessed, as in the rat, following an additional 28-day treatment-free period (Supplementary Fig. 5F). There were no decedents during this study, and all animals appeared to be healthy at the end of the treatment period. Transient clinical signs were observed during or immediately after dosing, intermittently throughout the study, with increased frequency and broader range with the higher dose (Fig. 6D). At the lower dose levels, there were only a few minor post-dosing observations. At 50 mg/kg/day, the most common clinical signs consisted of red body and/or ears, swollen ears, eyes and/or eyelids, mild body tremors, semi-closed eyes, and visible nictitating membrane (Fig. 6D). As in the rat, all clinical signs were rapidly reversible, generally resolving within 1 h of dosing and in all cases by the end of the day. In one animal investigated during the dose-ranging study, clinical signs were only marginally attenuated by prophylactic anti-histamine treatment with Phenergan, suggesting that they were not of an allergic nature (data not shown) ref [27]. Therefore, since all post-dosing observations were both rapidly and spontaneously reversible, and immunotoxicity evaluation is not required for the preclinical safety assessment of an oncological drug candidate, these effects were considered not to be dose limiting.

Apart from a transient and isolated increase in heart rate, which was within the normal range for the dog, at 50 mg/kg, there were no drug-related changes in electrocardiography (ECG) parameters, nor waveform morphology (data not shown). As in the rat, there were no treatment-related effects on body weight, food consumption, nor any haematology, clinical chemistry or urinalysis parameters at any dose level investigated, nor was there any drug-related ophthalmic change (Supplementary Fig. 5B; see list of in-life procedures). At the terminal kill, there were no treatment-related changes in organ weight, nor any macroscopic changes, except for a slightly increased incidence of red appearance at some injection sites (Supplementary Fig. 5G). Microscopically, this minor finding correlated with a mild drug-related increase in the incidence of fasciitis/fibrosis at two of the injection sites (Fig. 6E). There were no other microscopic findings which were suggestive of either a local or systemic effect of DTP3. Indeed, all other tissues were either histo-pathologically unremarkable or the findings were minor, infrequent and/or consistent with the typical pattern observed in animals of the same strain and age (Supplementary Fig. 5G). Following the recovery period, there was a complete reversal of the local findings at injection sites at the terminal kill (Fig. 6E). We concluded that, following repeated rapid daily infusion, DTP3 was tolerated in the dog at dose levels up to 50 mg/kg/day. Accordingly, the NOAEL of DTP3 in this species was considered to be the maximum administered dose of 50 mg/kg/day (Fig. 6F). Collectively, the methodical and extensive investigations conducted in the 28-day repeat-dose toxicity studies underscore the tolerability of DTP3 in both rodent and non-rodent species, with no target organs of toxicity, no preclusive adverse effect and no specific risk identified for the progression of this novel therapeutic into clinical evaluation in oncology.

### 2.8 Safety margins of exposure and starting clinical dose level

Based on the preclinical pharmacology and toxicology data and cancer-selective therapeutic mode of action of DTP3, we sought to initiate a phase I trial of this novel anticancer agent in patients with relapsed or refractory MM. To derive the clinical starting dose level and safety margin of exposure for this first-in-human study of DTP3, the rat was considered to be the most sensitive toxicological species, as based on the lowest values for unbound DTP3 fraction (Fig. 4B), the systemic exposure at the NOAEL in this species generated the most conservative clinical projections (Supplementary Fig. 3A–B). Upon allometric scaling by a conventional factor of six, the NOAEL in the rat corresponded to a human dose equivalent of 16.7 mg/kg, which was then reduced by a further factor of 4.2 to correct for differences in PPB between rat and man, using the most conservative values for each species (*i.e.*, 80% for rat and 19% for man; Fig. 4B), yielding a human dose equivalent for unbound drug fraction of approximately 4.0 mg/kg. An additional safety margin of eight folds was subsequently applied to obtain the clinical starting dose level of 0.5 mg/kg/day.

To estimate the safety margins of exposure relative to unbound DTP3, the mean area under the curve (AUC) values in rat and dog at the NOAEL were compared to the mean AUC derived in mouse at the therapeutically effective dose level of 10 mg/kg (Supplementary Fig. 3A-C; see also Fig. 5A-C). After correcting for inter-species differences in PPB, using either the most or least conservative values (Fig. 4B), the estimated safety margins of exposure relative to unbound drug ranged from approximately 5 to 15 folds in rat and 15 to 20 folds in dog, thus underscoring the wide preclinical therapeutic window of DTP3. Accordingly, the clinical protocol stipulates a potential dose escalation up to a maximal clinical dose of 20 mg/kg, corresponding to a 40-fold increment over the starting dose level. Indeed, as in the first-in-human phase I study, DTP3 is administered three times per week by a 1-hour infusion, rather than once every day by a 10-minute infusion, the risk to patients is likely to be significantly lower than in preclinical species, owing to the reduced dosing frequency and projected relative C_max_ values.

## 3 Discussion

Here, we report the safe and target-selective pharmacology of the first-in-class GADD45β/MKK7 inhibitor, DTP3. We show that DTP3 exhibits good tolerability, no target organs of toxicity and no preclusive adverse effects upon daily repeat-dose administration in either rodent or non-rodent species, resulting in a wide safety margin of exposure. DTP3 also displayed good drug-like properties and favourable PK and ADME profiles, suitable for administration by a clinically advantageous intravenous bolus schedule. Together with our previously published results [11], the current data demonstrate that the GADD45β/MKK7-targeting strategy to pharmacologically inhibit oncogenic NF-κB signalling succeeds in coupling safety with therapeutic anticancer efficacy, resulting in ablation of MM xenografts in mouse models and selective killing of primary human MM cells through a mechanism involving JNK-driven apoptosis, whilst producing no significant adverse effect and none of the dose-limiting toxicities of conventional IKKβ/NF-κB-targeting drugs [[2], [3], [4], [5], [6],^10^]. Collectively, these results underscore the cancer-selective pharmacology and clinical potential of DTP3 as a safe and effective anticancer therapeutic in patients with MM and potentially other NF-κB-driven cancers.

Interestingly, the initial results from the first-in-human phase I study of DTP3 in patients with relapsed/refractory MM confirmed the clinical capacity of this novel anticancer therapeutic to induce a cancer-selective pharmacodynamic response, triggering JNK signalling and apoptosis in MM, but not normal cells of patients, whilst producing no signs of toxicity and no adverse effects [28], thus providing clinical proof-of-concept for the novel approach to therapeutically block oncogenic NF-κB survival signalling.

Notably, while DTP3 combines favourable drug-like properties, including on-target specificity, long plasma half-life and good bioavailability, the safety and cancer-selective pharmacology of the therapeutic approach largely stem from the choice of the drug target and its biological properties. Crucially, in this respect, GADD45β displays tissue-restricted function and is dispensable for the survival of most normal cells [22,23]. Beyond these GADD45β-dependent properties, the therapeutic target is a protein-protein interaction, rather than a lone molecule, and both GADD45β and MKK7 play biological roles outside their involvement in the GADD45β/MKK7 complex and, significantly, retain these roles, including MKK7 enzymatic function, upon complex disruption by DTP3 [11]. This therapeutic mechanism enhances the selectivity of the approach, as well as its safety.

The positioning of the GADD45β/MKK7 survival module downstream in the NF-κB pathway further confers additional clinical advantages on DTP3, including its capacity to bypass MM-cell resistance to conventional anti-MM therapies, including proteasome inhibitors, IMiDs and steroids [11,[13], [14], [15]]. Indeed, due to this therapeutic efficacy in refractory cancer cells and its capacity to synergise with bortezomib, DTP3 represents a significant clinical opportunity for treating patients with relapsed or refractory MM.

Yet, while DTP3 produced no significant adverse effects in any of the animal species investigated, even when administered at elevated dose levels, there remain some unknowns relating its successful clinical translation into an effective GADD45β/MKK7-targeting therapeutic in oncology. Beyond the generic uncertainties associated with any novel drug candidate, an on-target-specific unknown in the clinical development of DTP3 relates to its potential pro-inflammatory adverse effects in patients. While producing no obvious phenotypes in unchallenged animals, genetic *Gadd45b* disruption was shown to enhance local inflammation and immune responses in experimental mouse models of arthritis, multiple sclerosis and oncogenesis [[29], [30], [31]]. Although in some cases, these effects appeared to be unrelated to the JNK pathway, in other cases, as in the context of arthritis, they were shown to depend on MKK7-driven JNK activation [29]. Therefore, DTP3 administration has the potential to exacerbate pro-inflammatory JNK signalling in patients with inflammatory or autoimmune comorbidities in which GADD45β/MKK7 is pathogenically involved. Even so, on the basis of all available data on DTP3 and results from the abovementioned genetic models, including the recent observation that myeloid-specific *Gadd45b* loss potentiates tumour-associated inflammation *via* a JNK-unrelated mechanism, any potential pro-inflammatory effects of DTP3 are expected to be both mild and localised, rather than systemic and dose-limiting, as in the case of IKKβ inhibitors [7,[32], [33], [34], [35]], and therefore most likely manageable with anti-inflammatory treatment. Similarly, the increased susceptibility of *Gadd45b^−/−^* mice to bacterial infections has been shown to involve a deregulation of the mitogen-activated protein kinase kinase kinase (MAPKKK), MAP/ERK kinase kinase 4 (MEKK4)/MAP three kinase 1 (MTK1) [36], rather than MKK7, and consequently would be unaffected by DTP3. Moreover, these potential risks associated with DTP3 administration represent a major step forward over the systemic dose-limiting toxicities of global IKKβ/NF-κB inhibition, and unlike these toxicities, would be acceptable for the development of a clinical drug candidate in oncology.

Future studies will determine the clinical safety and therapeutic efficacy of DTP3 in patients with MM and potentially other cancers in which NF-κB drives oncogenesis *via* GADD45β [31]. Notwithstanding, the preclinical safety and on-target-specific pharmacology of DTP3 we report here, together with the potent and cancer-selective therapeutic efficacy previously demonstrated in MM cells from patients and mouse MM xenograft models, and the recently published encouraging clinical results in MM patients [28], demonstrate that the safe inhibition of the NF-κB survival pathway is clinically achievable and capable of producing the desired therapeutic effects, with profound implications in oncology, beyond MM. Indeed, the basic principle of targeting a pathogenically critical axis of the NF-κB pathway, rather than NF-κB globally, and underpinning the development of DTP3, could be applied to safely target pathogenic NF-κB signalling also in GADD45β-independent malignancies and, perhaps, even non-malignant NF-κB-driven diseases.

## Authorship

L.T. and G.F. designed in-house experiments. L.T., G.A., J.E.C, J.K., N.A., S.B., and G.F. contributed to design experiments at external CROs. L.T., D.C., F.B., D.V., and J.B. performed experiments. L.T., D.D., G.A., L.E.C., J.K., M.T., N.A., H.E.O., M.F.K., A.W., J.F.A.; H.W.A. and G.F. analysed data. A.L., A.S., D.R., M.R., A.C., M.O., M.J.A., R.S. K., I.G., R.B., and H.W.A. contributed clinical samples or key reagents. L.T. and D.C. contributed to mouse studies. G.F. and L.T wrote the paper. D.C., D.D., and H.W.A. contributed to writing the paper.

## Financial support

This work was supported in part by Cancer Research UK programme grant A15115, Medical Research Council (MRC) Biomedical Catalyst grant MR/L005069/1 and Bloodwise project grant 15003 to G.F., and Cancer Research UK Clinician Scientist FellowshipC41494/A15448 to H.W.A. H.W.A. also acknowledges the support of the Imperial College London NIHR BRC, the Cancer Research UK Imperial Centre, and the Imperial Experimental Cancer Medicine Centre.

## Disclosure of potential conflict of interest

G.F., L.T. and M.R. are named inventors on patents relating to this research.

## Transparency document

Transparency document
