# Supplementary material for: Preclinical toxicology and safety pharmacology of the first-in-class GADD45β/MKK7 inhibitor and clinical candidate, DTP3
Source: Toxicol Rep. 2019 Apr 19;6:369–79. doi: 10.1016/j.toxrep.2019.04.006 (PMC6502747; doi:10.1016/j.toxrep.2019.04.006)

Figure S1

A

Reference compound	
**Receptors**	
1	A1 (h)
2	A2A (h)
3	A3 (h)
4	α1 (non selective)
5	α2 (non selective)
6	β1 (h)
7	β2 (h)
8	AT1 (h)
9	AT2 (h)
10	BZD (peripheral)
11	BB (non selective)
12	B2 (h)
13	CGRP (h)
14	CB1 (h)
15	CCK1 (CCKA) (h)
16	CCK2 (CCKB) (h)
17	D1 (h)
18	D2S (h)
19	D3 (h)
20	D4.4 (h)
21	D5 (h)
22	ETA (h)
23	ETB (h)
24	GABA (non selective)
25	GAL1 (h)
26	GAL2 (h)
27	PDGF
28	CXCR2 (IL-8B) (h)
29	CCR1 (h)
30	TNF-α (h)
31	H1 (h)
32	H2 (h)
33	MC4 (h)
34	MT1 (ML1A) (h)
35	M1 (h)
36	M2 (h)
37	M3 (h)
38	M4 (h)
39	M5 (h)
40	NK1 (h)
41	NK2 (h)
42	NK3 (h)

Reference compound	
**Receptors**	
43	Y1 (h)
44	Y2 (h)
45	NTS1 (NT1) (h)
46	δ2 (DOP) (h)
47	κ (KOP)
48	μ (MOP) (h)
49	NOP (ORL1) (h)
50	PAC1 (PACAP) (h)
51	PPARγ (h)
52	EP2 (h)
53	EP4 (h)
54	IP (PGI2) (h)
55	P2Y
56	5-HT1A (h)
57	5-HT1B
58	5-HT2A (h)
59	5-HT2B (h)
60	5-HT2C (h)
61	5-HT5a (h)
62	5-HT6 (h)
63	5-HT7 (h)
64	sigma (non selective) (h)
65	sst (non selective)
66	GR (h)
67	VPAC1 (VIP1) (h)
68	V1 a (h)
**Ion channels**	
69	BZD (central)
70	PCP
71	P2X
72	5-HT3(h)
73	Ca2+ channel (L, verapamil site)
74	KV channel
75	SKCa channel
76	Na+ channel (site 2)
77	Cl- channel (GABA-gated)
**Transporters**	
78	norepinephrine transporter (h)
79	dopamine transporter (h)
80	5-HT transporter (h)

B

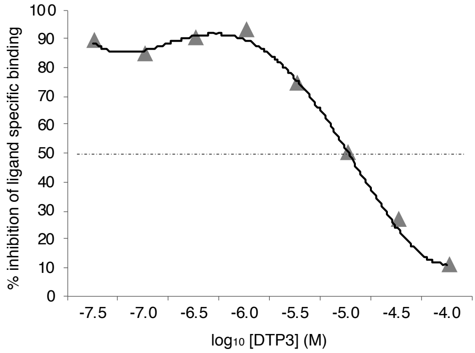

C

Sigma receptor (non selective)	IC_{50} (M)	K_i (M)
DTP3	1.3E-0.5	1.0E-0.5
haloperidol	6.8E-08	5.4E-0.8

D

Agonistic Effect		
	Response (% of ligand binding)	
[Drug] (M)	DTP3	(+) SKF-10,047
1.0E-0.6	0	-
1.0E-0.5	0	17
3.0E-0.5	-	48
1.0E-0.4	-3	116
**Antagonistic Effect**		
	Response (% of ligand binding)	
[Drug] (M)	DTP3	Rimcazole
1.0E-0.6	96	84
3.0E-0.6	-	49
1.0E-0.5	88	-6
1.0E-0.4	65	-

E

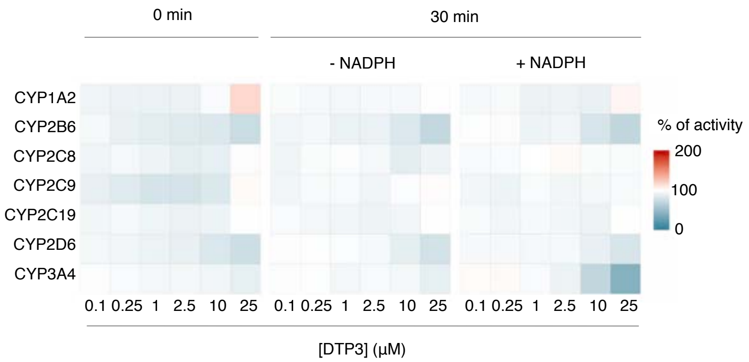

F

		0 min	30 min	
			- NADPH	+ NADPH
**Control compound**	**CYP isoform**	**IC_{50} (μM)**	**IC_{50} (μM)**	**IC_{50} (μM)**
furafylline	CYP1A2	9.64	9.14	0.405
thiotepa	CYP2B6	39.9	53.5	4.34
gemfibrozil glucuronide	CYP2C8	30.9	30.3	5.84
tienilic_acid	CYP2C9	2.32	2.58	0.3
fluoxetine	CYP2C19	18.5	22.5	5.17
paroxetine	CYP2D6	3.11	2.57	0.235
mifepristone	CYP3A4	6	6.05	0.942

Figure S2

A

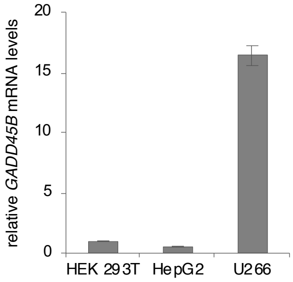

B

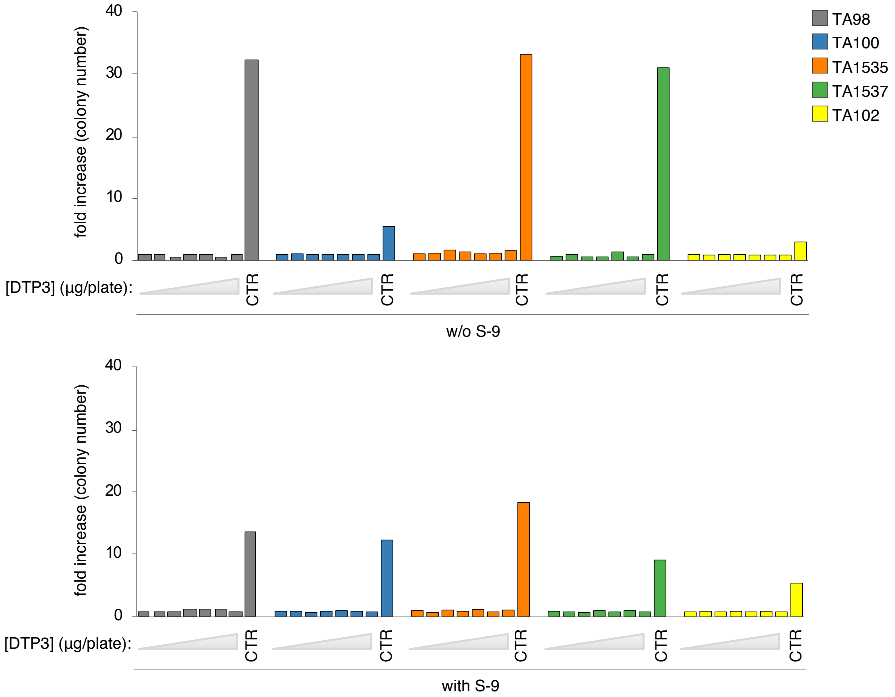

C

Control compaund	Concentration (µg/plate)	*S. Typhimurium* strain(s)	S-9
2-nitrofluorene (2NF)	5	TA98	-
Sodium azide (NaN_3)	2	TA100, TA1535	-
9-aminoacridine (AAC)	50	TA1537	-
Mitomycin C (MMC)	0.2	TA102	-
Benzo[a]pyrene (B[a]P)	10	TA98	+
2-aminoanthracene (AAN)	5	TA100, TA1535, TA1537	+
2-aminoanthracene (AAN)	20	TA102	+

Figure S3

A

Dose (mg/kg)	15		40		100	
	**day 1**					
	M	F	M	F	M	F
C_{max} (ng/ml)	37100	34600	79200	74500	163000	123000
$AUC_{(0-\infty)}$(ng*h/ml)	12900**	12800**	28800	36000	91800	88600
C_{max}/D (ng/ml)	2470	2300	1980	1860	1630	1230
$AUC_{(0-t)}$/D (ng*h/ml)	860	853	718	901	915	883
	**day 28**					
C_{max} (ng/ml)	45500	41800	83200	88800	198000	239000
$AUC_{(0-\infty)}$ (ng*h/ml)	14900	15500	29800	30000	81100	87100
C_{max}/D (ng/ml)	3030	2790	2080	2220	1980	2390
$AUC_{(0-t)}$/D (ng*h/ml)	989	1030	742	747	808	868
RA_{Cmax}	1.2	1.2	1.1	1.2	1.2	1.9
RA_{AUC}	1.2	1.2	1.0	0.8	0.9	1.0

B

Dose (mg/kg)	10		22		50	
	**day 1**					
	M	F	M	F	M	F
C_{max} (ng/ml)	26800	30400	59500	67300	199000	170000
$AUC_{(0-\infty)}$(ng*h/ml)	8430	9410	25200	24200	86200	71500
C_{max}/D (ng/ml)	2680	3040	2700	3060	3980	3390
$AUC_{(0-t)}$/D (ng*h/ml)	866	1010	1070	1110	1920	1670
	**day 28**					
C_{max} (ng/ml)	38300	32500	86400	85100	216000	209000
$AUC_{(0-\infty)}$ (n*h/ml)	11900	11100**	30900	32800	95000	98900
C_{max}/D (ng/ml)	3830	3250	3930	3870	4320	4170
$AUC_{(0-t)}$/D (ng*h/ml)	1190	1110	1390	1410	1870	1870
RA_{Cmax}	1.4	1.1	1.5	1.3	1.1	1.3
RA_{AUC}	1.4	1.1	1.3	1.3	1.0	1.2

C

Dose (mg/kg)	2.5	10	50
C_{max} (ng/ml)	3265	16345	130175
$AUC_{(0-t)}$ (ng*h/ml)	1029	4695	40538
C_{max}/D (ng/ml)	1306	1635	2604
$AUC_{(0-24hr)}$/D (ng*h/ml)	412	470	811

D

Dose (mg/kg)	2.5	10	50
C_{max} (ng/ml)	1580	6687	34875
$AUC_{(0-t)}$ (ng*h/ml)	1151	5392	39540
C_{max}/D (ng/ml)	632	669	698
$AUC_{(0-24hr)}$/D (ng*h/ml)	460	539	791

Figure S3

E

Tissue	*µg equivalents of DTP3/g of tissue* 10 min	1 hr	4 hr	24 hr	72 hr	168 hr
Plasma	16.6	3.74	0.086	0.034	0.033	0.032
Blood	9.09	2.06	0.111	0.095	0.035	0.032
Adrenal cortex	3.01	0.849	-	-	-	-
Adrenal medulla	4.01	1.01	-	-	-	-
Aortic wall	5.05	2.15	-	-	-	-
Bile ducts	68.6	48.2	2.11	0.730	0.659	-
Blood WBA	8.52	2.14	-	-	-	-
Bone marrow	2.92	1.11	-	-	-	-
Bone surface	1.27	7.44	-	-	-	-
Brain	-	-	-	-	-	-
Brown fat	2.91	0.777	-	-	-	-
Bulbo-urethral gland	5.50	1.81	-	-	-	-
Cartilage	16.7	5.72	0.522	-	-	-
Choroid plexus	1.95	1.79	1.30	0.404	0.647	0.344
Epididymis	1.44	0.581	-	-	-	-
Epimysium	3.27	0.930	-	-	-	-
Exorbital lachrymal gland	2.83	0.937	-	-	-	-
Harderian gland	1.98	0.922	-	-	-	-
Intra-orbital lachrymal gland	2.93	0.998	-	-	-	-
Kidney cortex inner	71.2	17.9	2.03	1.01	0.470	-
Kidney cortex outer	42.2	12.6	2.27	1.81	0.910	0.438
Kidney medulla	49.6	15.6	1.43	1.04	-	-
Lens	-	-	-	-	-	-
Liver	58.4	46.4	4.05	1.07	-	-
Lung	8.14	2.43	0.401	-	-	-
Ma-ibular lymph node	2.66	1.88	0.524	-	-	-
Meninges	2.91	0.459	-	-	-	-
Muscle	1.45	0.375	-	-	-	-
Myocardium	2.88	0.883	-	-	-	-
Nasal mucosa	1.90	1.12	-	-	-	-
Pancreas	4.15	4.78	3.18	0.819	-	-
Periodontal membrane	5.34	1.72	-	-	-	-
Pineal body	3.97	1.44	-	-	-	-
Pituitary	4.06	2.02	1.11	0.511	-	-
Preputial gla-	NS	4.41	4.39	1.85	-	-
Prostate	2.22	0.671	-	-	-	-
Salivary glands	3.46	1.54	-	-	-	-
Seminal vesicles	-	-	-	-	0.527	-
Skin	10.0	2.05	-	-	-	-
Spinal cord	-	-	-	-	-	-
Spleen	3.74	1.76	-	-	-	-
Testis	2.33	0.547	-	-	-	-
Thymus	1.89	2.39	0.391	-	-	-
Thyroid	4.23	1.55	0.367	-	-	-
Tongue	5.76	1.59	-	-	-	-
Tooth pulp	4.64	2.81	-	-	-	-
Trachea	5.50	1.54	0.608	-	-	-
Urinary bladder wall	32.0	31.4	0.704	0.778	-	-
Urine	536	205	17.0	1.51	-	-
Uveal tract/retina	7.34	2.78	0.613	-	-	-
White fat	2.26	-	-	-	-	-
Oesophageal wall	5.15	2.68	1.01	-	-	-
Stomach mucosa fundus	6.42	2.52	1.64	0.988	-	-
Stomach mucosa non fundic	6.57	3.60	-	1.66	-	-
Small intestine mucosa	4.64	4.17	-	2.91	-	-
Caecum mucosa	5.20	1.10	1.56	2.19	-	-
Large intestine mucosa	4.26	1.20	0.441	8.83	-	-
Rectum mucosa	9.52	2.86	-	0.631	-	-

F

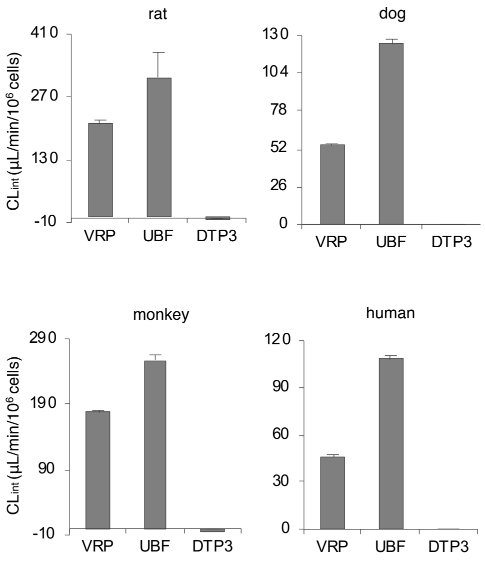

G

Region Identity	10 m	1 hr	4 hr	24 hr	72 hr	168 hr
P1	0.24	-	-	-	-	-
P2	0.4	-	-	-	-	-
P3	0.71	0.16	-	-	-	-
P4	1.06	1.23	-	-	-	-
P5	1.43	1.26	-	-	-	-
P6 (DTP3)	84.11	86.41	80.5	52.29	-	-
P7 (methoxy DTP3)	0.8	0.8	-	-	-	-
P8	0.32	0.37	-	-	-	-
P9 (oxidative deamination of DTP3)	9.23	7.69	4.99	-	-	-

Figure S4

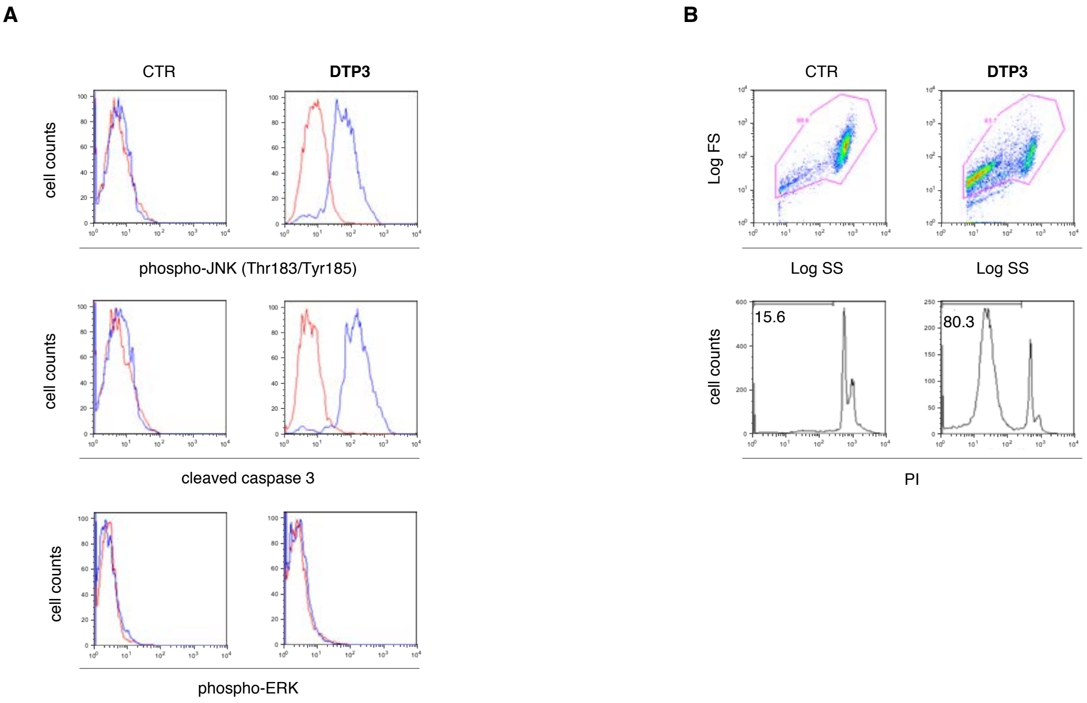
A
CTR
DTP3
cell counts
phospho-JNK (Thr183/Tyr185)
cell counts
cleaved caspase 3
cell counts
phospho-ERK
B
CTR
DTP3
Log FS
Log SS
Log SS
cell counts
15.6
80.3
PI

Figure S5

A

			Toxicity		Treatment-free (recovery)	
group	treatment	dose level (mg/kg/day)	M (n)	F (n)	M (n)	F (n)
1	control	0	10	10	5	5
2	low	15	10	10	-	-
3	intermediate	40	10	10	-	-
4	high	100	10	10	5	5
			Toxicokinetics[a]			
	treatment	dose level (mg/kg/day)	M (n)	F (n)		
1	control	0	3	3		
2	low	15	6	6		
3	intermediate	40	6	6		
4	high	100	6	6		

B

**Clinical Observations**
health monitoring
clinical examinations
post-dose observations
**Ophthalmic examinations**
**Body weights**
**Food Consumption**
**Electrocardiomiography***

**Clinical laboratory procedures**		
**Bone Marrow smear evaluation**		
**Clinical pathology**		
Haematology tests	***Clinical Chemistry tests***	***Urinalysis tests***
hemoglobin	Aspartate aminotransferase	volume
red blood cell count	Alanine aminotransferase	colour
packed cell volume	Alkaline phosphatase	turbidity
mean cell volume	γ-glutamyl transferase*	specific gravity
mean cell hemoglobin	total cholesterol	pH**
mean cell hemoglobin concentration	total bilirubin	protein**
reticulocyte count	total protein	glucose**
red cell distribution width	albumin	ketones**
hemoglobin distribution width	globulin	urobilinogen**
total and differential white cell count	albumin/Globulin ratio	bilirubin**
platelet count #	sodium	blood**
platelet crit	potassium	microscopy of sediment
mean platelet volume	chloride	
platelet distribution width	calcium	
Coagulation tests	inorganic phosphate	
prothrombin time	creatinine	
fibrinogen	urea	
activated partial thromboplastin time	glucose	

Figure S5

C

		Terminal kill								Recovery kill			
		CTR		DTP3 (mg/kg/day)						CTR		DTP3 (mg/kg/day)	
				15		40		100				100	
		M (n=10)	F (n=10)	M (n=9)	F (n=10)	M (n=10)	F (n=9)	M (n=9)	F (n=10)	M (n=5)	F (n=5)	M (n=5)	F (n=4)
**Clinical Chemistry**													
Sodium (mmol/L)		141 ± 1.1	141± 1.4	142 ± 0.7	141 ± 1.1	141 ± 1.4	141 ± 1.0	142 ± 1.1	142 ± 0.9	140 ± 1.2	140 ± 1.8	140 ± 1.3	142 ± 0.8
Potassium (mmol/L)		3.8 ± 0.23	3.3 ± 0.21	3.9 ± 0.17	3.5 ± 0.19	4.0 ± 0.21	3.6 ± 0.15	3.7 ± 0.27	3.5 ± 0.21	3.9 ± 0.26	3.3 ± 0.19	3.7 ± 0.12	3.6 ± 0.17
Chloride (mmol/L)		100 ± 1.3	100 ± 1.3	101 ± 0.8	101 ± 0.7	100 ± 1.3	100 ± 1.1	100 ± 0.7	100 ± 1.2	101 ± 1.9	100 ± 2.3	101 ± 1.0	102 ± 1.0
Calcium (mmol/L)		2.6 ± 0.07	2.59 ± 0.05	2.6 ± 0.09	2.63 ± 0.05	2.7 ± 0.08	2.64 ± 0.06	2.6 ± 0.05	2.61 ± 0.07	2.53 ± 0.08	2.55 ± 0.05	2.53 ± 0.06	2.66 ± 0.07
Inorganic Phosphate (mmol/L)		2.3 ± 0.17	2 ± 0.26	2.2 ± 0.28	2.1 ± 0.16	2.2 ± 0.16	1.9 ± 0.21	2.1 ± 0.17	2 ± 0.36	1.7 ± 0.26	1.9 ± 0.15	1.6 ± 0.11	2.0 ± 0.29
Enzymatic creatinine (mmol/L)		23 ± 2.7	28 ± 1.8	24 ± 3.1	28 ± 3.7	25 ± 1.8	27 ± 3.4	24 ± 2.4	27 ± 2.4	28 ± 2.3	31 ± 2.9	28 ± 2.0	26 ± 2.5
Urea (molo/L)		7.6 ± 0.88	7.9 ± 0.98	7.2 ± 1.02	8.4 ± 0.81	6.8 ± 0.97	8.5 ± 0.89	7.3 ± 1.31	7.2 ± 1.29	7.3 ± 1.03	8.3 ± 0.99	7.1 ± 0.71	7.7 ± 1.35
**Urinalysis**													
Volume (mL)		3.6 ± 1.19	2.6 ± 1.02	4.9 ± 0.91	2.7 ± 0.87	5.6 ± 1.32	3.1 ± 1.4	3.7 ± 1.55	4.0 ± 1.18	4.7 ± 1.76	2.5 ± 1.02	3.6 ± 2.38	2.3 ± 1.26
Specific Gravity		1.03 ± 0.006	1.035 ± 0.008	1.027 ± 0.004	1.035 ± 0.009	1.025 ± 0.005	1.034 ± 0.007	1.035 ± 0.011	1.031 ± 0.005	1.036 ± 0.009	1.042 ± 0.013	1.043 ± 0.016	1.053 ± 0.025
PH		7 ±0.57	6.2 ± 0.42	7 ± 0.35	6.45 ± 0.59	7.1 ± 0.46	5.94 ± 0.39	6.4 ± 0.39	6 ± 0.23	7 ± 0.35	6.3 ± 0.44	7.1 ± 0.41	6.37 ± 0.47
Colour	pale straw	8	9	7	10	9	9	4	9	0	0	0	0
	straw	2	1	2	0	1	0	5	1	5	5	5	4
Turbidity	clear	9	10	3	8	6	9	8	10	4	5	4	4
	hazy	1	0	6	2	4	0	1	0	1	0	1	0
	cloudy	0	0	0	0	0	0	0	0	0	0	0	0
Protein (g/L)	negative	0	2	0	0	0	2	0	6	0	0	0	0
	trace	2	5	4	6	4	7	4	4	1	2	0	2
	0.3-0.9	6	3	5	3	6	0	4	0	3	3	3	1
	1-2.9	2	0	0	1	0	0	1	0	1	0	2	1
	3-20+	0	0	0	0	0	0	0	0	0	0	0	0
Glucose (mmol/L)	negative	10	10	9	10	10	9	9	10	5	5	5	4
	trace	0	0	0	0	0	0	0	0	0	0	0	0
	5.5-14	0	0	0	0	0	0	0	0	0	0	0	0
	15-28	0	0	0	0	0	0	0	0	0	0	0	0
	29-55	0	0	0	0	0	0	0	0	0	0	0	0
Ketones (mmol/L)	negative	0	8	2	9	3	9	1	10	0	5	0	4
	trace	8	2	6	1	7	0	8	0	5	0	2	0
	0.5-1.5	2	0	1	0	0	0	0	0	0	0	3	0
	1.6-4	0	0	0	0	0	0	0	0	0	0	0	0
	4.1-8	0	0	0	0	0	0	0	0	0	0	0	0
Urobilinogen (μmol/L)	3.2	10	10	9	10	10	9	9	10	5	5	5	4
	3.3-16	0	0	0	0	0	0	0	0	0	0	0	0
	16.1-33	0	0	0	0	0	0	0	0	0	0	0	0
	33.1-66	0	0	0	0	0	0	0	0	0	0	0	0
	66.1-131+	0	0	0	0	0	0	0	0	0	0	0	0
Bilirubin	negative	9	10	9	10	10	9	8	10	3	5	3	3
	1+	1	0	0	0	0	0	1	0	2	0	2	1
	2+	0	0	0	0	0	0	0	0	0	0	0	0
	3+	0	0	0	0	0	0	0	0	0	0	0	0
Blood	negative	9	10	8	9	9	9	7	9	5	5	5	4
	trace	1	0	0	1	0	0	2	1	0	0	0	0
	1+	0	0	1	0	1	0	0	0	0	0	0	0
	2+	0	0	0	0	0	0	0	0	0	0	0	0
	3+	0	0	0	0	0	0	0	0	0	0	0	0
Red Blood Cells (cells/field)	0	10	10	9	10	10	9	9	10	5	5	4	4
	1-10	0	0	0	0	0	0	0	0	0	0	1	0
	11-30	0	0	0	0	0	0	0	0	0	0	0	0
	>30	0	0	0	0	0	0	0	0	0	0	0	0
White Blood Cells (cells/field)	0	10	10	9	8	10	9	7	7	5	5	5	3
	1-10	0	0	0	1	0	0	1	1	0	0	0	1
	11-30	0	0	0	0	0	0	1	1	0	0	0	0
	>30	0	0	0	1	0	0	0	1	0	0	0	0
Epithelial Cells (cells/field)	0	8	3	8	3	7	3	5	2	2	1	1	0
	1-10	2	7	1	6	3	6	4	8	3	4	4	4
	11-30	0	0	0	1	0	0	0	0	0	0	0	0
	>30	0	0	0	0	0	0	0	0	0	0	0	0
Sperm	negative	5	10	6	10	7	9	4	10	2	5	1	4
	scanty	4	0	0	0	3	0	3	0	0	0	2	0
	some	1	0	3	0	0	0	2	0	2	0	2	0
	heavy	0	0	0	0	0	0	0	0	1	0	0	0
Casts (casts/field)	0	10	10	9	10	10	9	9	10	5	5	5	4
	1-5	0	0	0	0	0	0	0	0	0	0	0	0
	6-10	0	0	0	0	0	0	0	0	0	0	0	0
	>10	0	0	0	0	0	0	0	0	0	0	0	0
Phosphates crystals	negative	0	7	0	6	1	7	3	10	0	1	1	1
	scanty	0	0	0	1	1	2	0	0	0	2	0	1
	some	4	3	0	1	1	0	5	0	0	2	0	0
	heavy	6	0	9	2	7	0	1	0	5	0	4	2
Urate Crystals	negative	10	10	9	10	10	9	9	10	5	5	5	4
	scanty	0	0	0	0	0	0	0	0	0	0	0	0
	some	0	0	0	0	0	0	0	0	0	0	0	0
	heavy	0	0	0	0	0	0	0	0	0	0	0	0
Calcium Oxalate Crystals	negative	10	10	9	10	10	9	9	10	5	4	5	4
	scanty	0	0	0	0	0	0	0	0	0	1	0	0
	some	0	0	0	0	0	0	0	0	0	0	0	0
	heavy	0	0	0	0	0	0	0	0	0	0	0	0
Uric Acid Crystals	negative	10	10	9	10	10	9	9	10	5	5	5	4
	scanty	0	0	0	0	0	0	0	0	0	0	0	0
	some	0	0	0	0	0	0	0	0	0	0	0	0
	heavy	0	0	0	0	0	0	0	0	0	0	0	0
Amorphous Debris	negative	0	0	3	0	2	0	0	0	0	0	0	0
	scanty	9	5	6	4	7	1	6	4	4	2	2	1
	some	1	5	0	6	1	7	3	5	1	3	2	3
	heavy	0	0	0	0	0	1	0	1	0	0	1	0
Bacteria	negative	1	4	4	2	3	1	0	2	5	5	5	4
	scanty	1	3	2	3	1	3	1	2	0	0	0	0
	some	2	0	2	2	3	5	3	3	0	0	0	0
	heavy	6	3	1	3	3	0	5	3	0	0	0	0

Figure S5

D

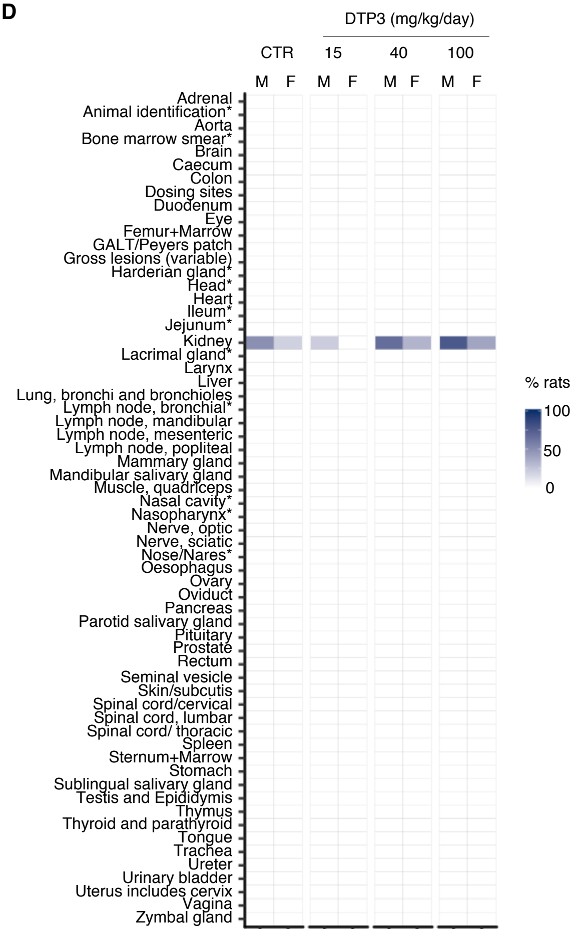

F

group	treatment	dose level (mg/kg/day)	Toxicity M (n)	Toxicity F (n)	Treatment-free (recovery) M (n)	Treatment-free (recovery) F (n)
1	control	0	3	3	2	2
2	low	10	3	3	-	-
3	intermediate	22	3	3	-	-
4	high	50	3	3	2	2

G

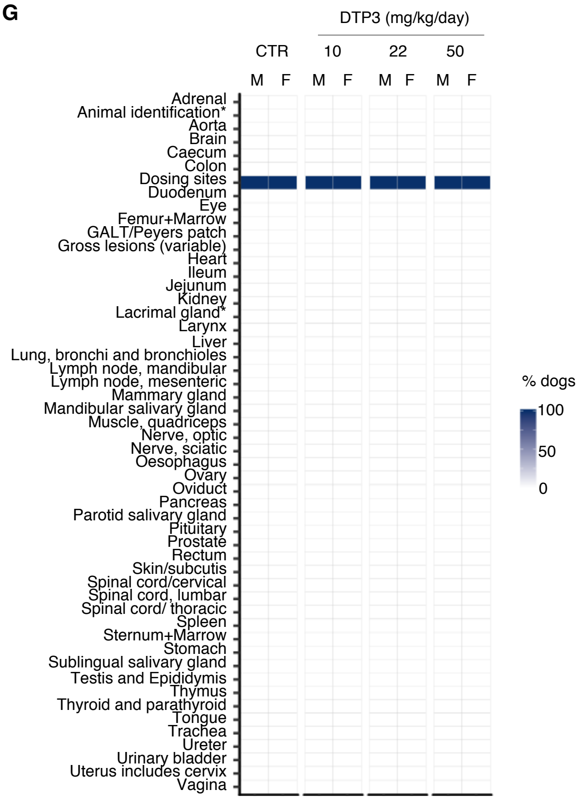

E

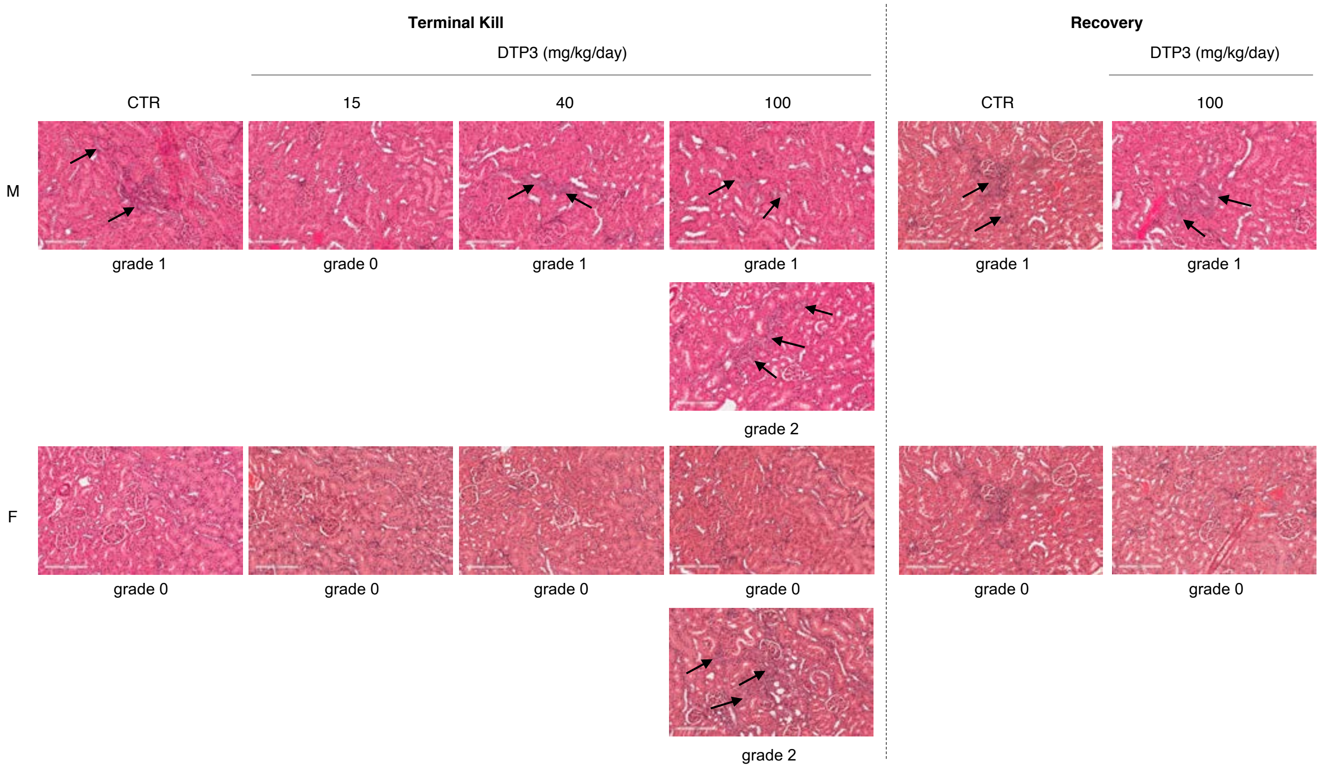

Supplement: Supplementary file 2 [file mmc2.pdf]
