# Supplementary material for: Preclinical toxicology and safety pharmacology of the first-in-class GADD45β/MKK7 inhibitor and clinical candidate, DTP3
Source: Toxicol Rep. 2019 Apr 19;6:369–79. doi: 10.1016/j.toxrep.2019.04.006 (PMC6502747; doi:10.1016/j.toxrep.2019.04.006)
Supplement: Supplementary file 3 [file mmc3.pdf]

SUPPLEMENTARY MATERIAL

SUPPLEMENTARY METHODS

Mouse Xenograft Studies

Mice were housed in the animal facilities at Hammersmith campus. All experiments were conducted under the authority of UK Home Office project license, P75F16A53, following approval by the Imperial College Ethical Review Process, and were performed essentially as previously described (1). Briefly, 6- to 8-week old NOD/SCID male mice (NOD.CB17-*Prkdc*^{scid}/IcrCrl; Charles River) were treated by intraperitoneal injection of anti-CD122 monoclonal antibody and, 24 hours later, subcutaneously injected with 1.0x10^7 human U266 MM cells re-suspended in 200 µL of sterile 1x phosphate buffered saline (PBS) and Matrigel (1:1). After ten days, mice were randomised into treatment groups and treated intravenously with saline solution (vehicle) or 10 mg/kg of DTP3 according to the indicated administration schedules. Animals were monitored daily and humanely killed at the indicated endpoints. Tumour volumes were measured using a calliper, and tumour cells were analysed as previously described (1). The U266 MM cell line was purchased from the Istituto Zooprofilattico "Bruno Umbertini" (Brescia, Italy) and cultured as previously described (1).

Flow Cytometric Analysis

To assess the PD response to DTP3, *in vivo*, U266 cells were purified from MM xenografts using CD138 MicroBeads (Miltenyi Biotech), according to the manufacturer's instructions, and then stained with the following antibodies: Alexa Fluor 647-conjugated phospho-JNK antibody, Alexa Fluor 647-conjugated isotype control antibody, PE-conjugated phospho-ERK antibody, PE-conjugated isotype

control antibody, Alexa Fluor 488-conjugated cleaved caspase-3 antibody, and Alexa Fluor 488-conjugated isotype control antibody (Cell Signalling). Briefly, cells were spun down, washed and fixed by adding Fix Buffer I (BD Biosciences) and then incubated at 37°C for 10 minutes. Following incubation, cells were washed with 1x PBS, permeabilised by resuspending them in Perm Buffer (BD Biosciences) with vigorous vortexing, and then temporarily stored at -20°C. Permeabilised cells were subsequently washed with 1x PBS and stained by incubating them with the indicated antibodies at room temperature in the dark for 40 minutes. Cells were finally washed with 1x PBS, acquired using a Cyan flow cytometer (Becton Dickinson), and analysed with FlowJo software.

Apoptosis and Cell Viability Assays

Analyses of apoptosis were performed using propidium iodide (PI) nuclear labelling assays, as previously described (1,2). Briefly, tumours were minced using a scalpel and incubated at 37°C for 40 minutes with collagenase D (5 mg/mL; Roche) in 5 mL of complete RPMI-1640 medium, as previously described (1). Single cell suspensions were prepared by passing the digested tumour tissue through a 70-μm cell strainer. Cells were then washed with 1x PBS and treated with ribonuclease A (50 μg/mL) at room temperature for 30 minutes. Cells from MM xenografts were finally stained with PI (50 μg/mL) in 0.1% sodium citrate buffer at 4°C in the dark for 2 to 4 hours, acquired using a Cyan flow cytometer (Becton Dickinson) and analysed with FlowJo, as previously described (1,2). Apoptotic cells were identified by the presence a sub-G_1 DNA content. MM cell viability in tumour samples was also evaluated by performing trypan blue exclusion assays, using anti-CD138 antibody to identify MM cells. Approximately $5.0x10^5$ cells were stained with FITC-conjugated anti-CD138 antibody (BD Biosciences), and analysed by flow cytometry using a

Beckman Coulter CyAn instrument. The percentages of $CD138^+$ U266 MM cells in tumour samples were assessed with FlowJo.

Quantitative Real-Time Polymerase-Chain Reaction (qRT-PCR)

Total RNA was extracted with Trizol (Invitrogen) and purified using the Direct-zol RNA microPrep kit (Zymo Research), according to the manufacturer's instructions. 1 µg of RNA was added as template to reverse-transcriptase (RT) reactions, performed using the High-Capacity cDNA Reverse Transcription Kit (Applied Biosystems), according to the manufacturer's instructions. qRT-PCR reactions were carried out as previously described (1). Experimental ΔΔCt values were normalised to glyceraldehyde 3-phosphate dehydrogenase (GAPDH), and relative mRNA levels were calculated against a reference sample (*i.e.*, HEK-293T-cell mRNA).

Radio-ligand Binding Assays

The secondary pharmacology of DTP3 was investigated, *in vitro*, using radio-ligand competition binding assays performed at CEREP laboratories in accordance with in-house standard operating procedures (SOP) (http://www.cerep.fr/cerep/users/pages/ProductsServices/InVitro.asp). Briefly, DTP3 was evaluated at the concentration of 10 µM against the panel of 80 validated drug targets listed in Supplementary Figure 1A (Figure 1A). According to CEREP's SOP, competitive inhibition greater than 50% was considered to be a significant effect and as such used as cut-off value for conducting further analyses. Consequently, the binding affinity of DTP3 for Sigma receptors (non-selective) was investigated in dose-response binding assays, testing concentrations from 30 nM to 100 µM and using haloperidol as a reference compound (Supplementary Figure 1B-C).

CYP Inhibition and Induction Assays

The potential of DTP3 for mediating drug-drug interactions via CYP isoforms was investigated *in vitro*, using time-dependent inhibition and catalytic induction assays conducted at Cyprotex laboratories in accordance with in-house SOP (http://www.cyprotex.com/admepk/). Briefly, for time-dependent inhibition assays, the effects of DTP3 on CYP1A2, CYP2B6, CYP2C8, CYP2C9, CYP2C19, CYP2D6 and CYP3A4 were evaluated in human liver microsomes at drug concentrations ranging from 0.1 µM to 25 µM, in the presence or absence of a 30-minute pre-incubation with or without NADPH, as shown, by assessing the formation of isoform-specific metabolites. IC_{50} values were calculated under each condition by determining the concentrations of DTP3 resulting in a 50% inhibition of metabolite formation (Figure 1B). The positive controls used in the assays are listed in Supplementary Figure 1F. For catalytic induction assays, the effects of DTP3 on CYP3A4, CYP2B6 and CYP1A were investigated in primary human hepatocytes upon daily treatment with DTP3 at the concentrations of 0.1, 1.0 and 10 µM for 72 hours. The formation of isoform-specific metabolites was quantified using LC-MS(/MS) or fluorescence analysis, as needed, upon addition of the following isoform-specific substrates: midazolam (20 µM) for CYP3A4; bupropion (50 µM) for CYP2B6; ethoxyresorufin (20 µM) for CYP1A. Isoform-specific control inducers were as follows: omeprazole (O) for CYP1A; phenobarbital (P) for CYP2B6; rifampicin (R) for CYP3A4 (Figure 1C). The fold induction for each isoform was determined by comparing the levels of isoform-specific metabolites following treatment with DTP3 or control inducers versus treatment with vehicle control (Figure 1C).

Bacterial Reverse Mutation Assays

The mutagenic potential of DTP3 was investigated, *in vitro*, in a GLP-compliant bacterial reverse mutation assay (Ames test) conducted at Covance laboratories in accordance with in-house SOP (https://www.covance.com/services/safety-

assessment/genetic-toxicology/regulatory-assays.html). Briefly, the numbers of revertant colonies for each of five histidine-requiring strains of *Salmonella typhimurium* (*i.e.*, TA98, TA100, TA1535, TA1537 and TA102), each reporting on different classes of genetically active compounds, were evaluated either in the presence or absence of metabolic activation by Aroclor 1254-induced rat liver post-mitochondrial S9 fraction (Supplementary Figure 2B). DTP3 was tested at the concentrations of 5, 16, 50, 160, 500, 1600 and 5000 μg/plate. Results were expressed as the mean fold increase in colony number for each tester strain upon exposure to DTP3 or strain-specific control compounds (CTR) relative to exposure to vehicle control, either in the presence or absence of metabolic activation (Supplementary Figure 2C). The following significance thresholds were applied to each strain: ≥1.5 folds, strain TA102; ≥2-fold, strains TA98 and TA100; ≥3-fold, strains TA1535 and TA1537.

Cytotoxicity Assays

The cytotoxicity of DTP3 was investigated in a multi-parametric *in-vitro* assay, performed at Cyprotex laboratories in accordance with in-house SOP (http://www.cyprotex.com/toxicology/multiparametric/cytotoxicity-screening-panel), using High Content Screening (HCS). Briefly, HepG2 cells were seeded onto wells of 96-well plates and, 24 hours later, treated with DTP3 or positive control compounds (*i.e.*, carbonyl cyanide and chlorpromazine), at the concentrations of 0.04, 0.1, 0.4, 1.0, 4.0, 10, 40, and 100 μM. Following a 72-hour incubation, cells were treated with a specific dye or antibody, as required by the assay. Plates were then scanned using an ArrayScan VTI automated fluorescence cell imager (Thermo Scientific Cellomics) and analysed for the following measures of toxicity: nuclear size and nuclear morphology, cell membrane permeability, mitochondrial mass, mitochondrial membrane potential ($\Delta\Psi$m), and cytochrome *c* release (Figure 2). For

each compound, cytotoxicity was scored by assessing the minimum effective concentration (MEC) inducing a significant effect relative to vehicle control.

***In-vitro* hERG Assays**

The effects of DTP3 on the hERG potassium ion channel were investigated in a GLP-compliant study conducted *in vitro* at B'SYS Laboratories in accordance with in-house SOP (http://www.bsys.ch/services/cardiac-panel/herg.html), using the whole-cell patch-clamp technique. DTP3 was tested at the concentration of 150 µg/mL in HEK-293 cells stably expressing ectopic hERG (NCBI Reference: NM_000238). Vehicle (0.1% DMSO) and the I_{Kr} blocker, E-4031 (100 nM) were used as negative and positive controls, respectively. Briefly, upon formation of a stable Gigaohm (GΩ) seal between patch electrodes and individual hERG-expressing HEK-293 cells, hERG-dependent outward tail currents were measured following depolarisation of the cell membrane to +2.0 mV for 2 s (activation of channels) from a holding potential of -80 mV, and following subsequent repolarisation to -40 mV, for 3 s. This voltage protocol was run at least 10 times at intervals of 10 s. Once control recordings had been taken, cells were continuously perfused with a bath solution containing DTP3 (150 µg/mL), DMSO (0.1%) or E-4031 (100 nM). Electrophysiological measurements were recorded using PatchMaster software (Version v2x73_2). The recorded current amplitudes at the steady state level of current inhibition were compared to those from control conditions, measured in the pre-treatment phase of the same cell, using SigmaPlot 11.0 software. The amount of current block was calculated as the percentage of control current. To determine whether the measured current inhibition was due to an interaction of the compounds with the hERG channel or to current rundown, residual currents were compared to those measured in DMSO-treated cells. Data from at least three experiments were collected for each compound, and the corresponding mean values and standard deviation (SD) were subsequently

determined. As treatment with DTP3 at 150 μg/mL produced no significant inhibition of hERG-dependent tail currents relative to vehicle-treated cells, no concentration-response curves, nor resulting IC_{50} values were determined.

Safety Pharmacology Studies

The GLP-compliant safety pharmacology studies of DTP3 were conducted at Covance Laboratories in accordance with in-house SOP (https://www.covance.com/services/safety-assessment/safety-pharmacology-services.html). The study design was based on the principles of the ICH Guidelines (Topic S7A; CPMP/ICH/539/00) on Safety Pharmacology Studies for Human Pharmaceuticals (November 2000). In all studies, DTP3 was formulated in 40 mM phosphate buffer pH 7.0 at the concentration of 80 mg/mL. Both vehicle and DTP3 were administered *in vivo* by a 10-minute intravenous infusion. All procedures conducted on live animals were subject to the provisions of United Kingdom Law, in particular the Animals (Scientific Procedures) Act, 1986.

The effects of DTP3 on the CNS were assessed according to Irwin's test (3), evaluating autonomic and locomotor activity, general behaviour, and body temperature in freely moving conscious rats. Prior to the study, 6-week-old male rats (Han Wistar; Charles River) were acclimatised for at least 5 days. Animals then were randomised into treatment groups (n=6), treated by single intravenous infusion of vehicle or DTP3 at the dose levels of 15, 40 and 100 mg/kg, and finally assessed according to the parameters listed in Figure 3H. Drug-induced changes were scored by using increasing integers, with '0' being normal (+/-, present/absent also being used). Parameters that were also measurable in normal animals were scored using an integer that allowed for both increases or decreases to be recorded. Observations were performed pre-dosing, immediately after dosing, and at 30, 60, 180 and 240 minutes post-dosing. Body temperature was measured as the rectal

temperature. For these measurements, animals were securely and calmly held by a competent operator whilst a lubricated digital thermometer probe was inserted (approximately 2 cm) into the rectum of the rat. All body temperature measurements were recorded pre-dosing and immediately after each Irwin's observation.

The risk assessment of DTP3 on the respiratory system was conducted in conscious, freely moving rats using whole-body plethysmography. The plethysmograph system consisted of individual chambers (one per animal) and a top reference chamber for filtering out changes in atmospheric pressure. Differential pressure transducers measured the pressure difference between the main and the reference chambers, thus eliminating any ambient pressure changes from the true flow signals. The temperature and relative humidity within each chamber were measured, and these measurements were used to compensate the flow signals, thus increasing the accuracy of the measured tidal volumes. Each whole-body plethysmograph chamber was linked to an 'eDacq' (EMMS data acquisition) system for recording respiratory parameters. 6-week-old male rats (Han Wistar; Charles River) were acclimatised in their own plethysmograph boxes for approximately 1 hour. The mean of the recordings from two pre-dosing intervals was calculated and reported. Rats were then removed from plethysmograph boxes, randomised into four groups (n=6) and then treated by single intravenous infusion of vehicle or DTP3 at the dose levels of 15, 40 and 100 mg/kg. After dosing, each rat was returned to its designated plethysmograph box, and respiratory parameters were subsequently measured and recorded. The mean readings over each 5-minute interval for the first 30 minutes post-dosing, and any subsequent consecutive 30-minute intervals for up to 6 hours post-dosing were calculated and reported. Both the rate and depth of respiration were measured, monitoring tidal volume, rate of respiration and minute volume, as reported in Figure 3E-G.

The effects of DTP3 on the cardiovascular system were evaluated in conscious, freely moving dogs, using radio-telemetry. Briefly, calibrated sensors (DSI D70 Series) for monitoring arterial blood pressure (ABP) and Lead II electrocardiogram (ECG) were surgically implanted into four male dogs (pure-bred beagle; Harlan). Following treatment for endoparasites and a course of vaccinations, animals were acclimatised for at least a week. A veterinary inspection was performed before the start of the procedures to ensure the suitability of the animals for the study. On the first day of dosing, animals weighed 10.17 to 15.24 kg and were approximately 33 to 38 months of age. Control of bias was based on the use of the Latin square design. Each animal received a single administration by intravenous infusion of either vehicle or DTP3 at the dose level of 10, 17 or 25 mg/kg. The dosing regimen was designed to ensure that all dose levels were represented on each dosing day, that each animal received each dose only once, and that every animal received a unique dosing sequence. A washout interval of at least 7 days occurred between doses. Formal behavioural observations were made approximately 1, 2, and 4 hours post-dosing. On each dosing day, ECG and blood pressure measurements were recorded for nominally 1 hour before dosing and continuously for up to 24 hours post-dosing. Two pre-dosing readings were taken 15 minutes apart (nominally 20 and 5 minutes) before administration of each dose. After start of the infusion, readings were taken from line averages at 5 and 10 minutes (*i.e.*, during and at the end of the infusion), 15 and 45 minutes (*i.e.*, post infusion in sling), then hourly until 12 hours post-dosing, and subsequent to this, once every 2 hours until 24 hours post-dosing. Data Sciences International (DSI) Dataquest® OpenART® radio-telemetry equipment was used both to generate and acquire the data. The system transferred the data to an online PoNeMah P3P (Ponemah Physiology Platform) digital acquisition and analysis system. Transmitters and amplifiers were switched on remotely in order to commence data generation.

Each recording pen was fitted with up to four DSI receivers, which were multiplexed together to act as one receiver. Corrected QT (QTc) intervals were calculated using Fridericia's correction.

Toxicology Studies

The GLP-compliant 28-day repeat-dose toxicity studies of DTP3 were conducted in rat (Crl:WI Han strain; Charles River) and dog (Beagle strain; Harlan) at Covance Laboratories according to in-house SOP (https://www.covance.com/services/lead-optimization/lead-optimization-non-glp-toxicology/safety-assessment.html). The rat and dog were selected for this study of DTP3 because they are the rodent and non-rodent species of lowest neurophysiological sensitivity known to be accepted by regulatory authorities, and *in vitro* metabolism studies in hepatocytes had indicated that there was no metabolism in human, monkey, rat or dog (Supplementary Figure 3F). The study design was based on ICH guidelines ICH M3 R2 and ICH S9. In each species, DTP3 was first administered intravenously by a 10-minute infusion at ascending dose levels to establish the maximum tolerated dose (MTD), and then given daily to naïve animals at the MTD for at least seven days to assess the toxicity of repeat-dose administration. As part of these dose-ranging studies, DTP3 was administered at doses up to 150 mg/kg in rat and up to 75 mg/kg in dog, and the MTD values in these species were determined to be 100 mg/kg and 50 mg/kg, respectively (Figure 6C, Figure 6F). These doses levels were subsequently selected as the high doses for the 28-day repeat-dose toxicity studies. The low dose levels of 15 mg/kg in rat and 10 mg/kg in dog were chosen based on PK studies as small multiples of the therapeutic doses. The intermediate dose levels (*i.e.*, 40 mg/kg in rat; 22 mg/kg in dog) were selected as the approximate geometric means of the low and high doses. 6- to 7-week-old rats of the Crl:WI Han strain (Charles River) and 4- to 5-month old dogs of the Beagle strain (Harlan) were individually identified by an

electronic implant and treated according to the protocols shown in Supplementary Figure 5A and Supplementary Figure 5F, respectively. DTP3 was formulated in 40 mM phosphate buffer pH 7.0 at the concentration of 80 mg/mL.

Animals were dosed by intravenous infusion into a lateral tail vein (rat) or a cephalic or saphenous vein (dog), once every day for 28 days, excluding the day of the necropsy. Each animal was given a detailed physical examination daily during the dosing period and once a week during non-dosing periods, at the beginning and the end of the working day for signs of ill health or toxicity. All animals were observed at the end of the infusion and approximately 1 hour after the end of infusion. Additional observations were made at the end of the day as required. Further in-life procedures consisted of an ophthalmic examination performed on all animals, pre-treatment and during week 4. Individual body weights were recorded before treatment on the first day of dosing, then at weekly intervals, and then again before necropsy. The amount of food consumed by each animal was determined once a day. Food consumption was calculated as g/animal/day. An ECG evaluation was conducted on all dogs in the main toxicity study, pre-treatment and during week 4, shortly after the end of the infusion. ECG analyses were also conducted on all treatment-free dogs, pre-treatment and during week 4 of both the dosing and recovery phases of the study. For clinical laboratory procedures, including haematology, coagulation and clinical chemistry tests, blood samples were withdrawn from the jugular vein of all animals, pre-treatment, during week 4, and at the end of the treatment-free period. Samples were collected before feeding. For urinalysis tests, urine samples were collected directly via a catheter from all animals, pre-treatment, during week 4, and at the end of the treatment-free period. A summary of the in-life procedures and laboratory tests conducted in both rat and dog is presented in Supplementary Figure 5B.

For pathological examination, scheduled necropsies were performed during week 4 of both the dosing and recovery phases of the study, after an overnight period without food. Animals were weighed before necropsy. Organs were dissected from all animals and weighed free from fat and other contiguous tissue before fixation. Left and right organs were weighed together. A full macroscopic examination of organs and tissues was performed under the general supervision of a pathologist and all lesions were recorded. Appropriate tissue samples from each animal were preserved in neutral-buffered 10% formalin, embedded in paraffin wax BP (block stage), sectioned at a nominal 5 µm, stained with haematoxylin and eosin, and finally examined microscopically by the study pathologist. A summary of the organs and tissue examined macroscopically and/or microscopically as part of the terminal procedures conducted in rat and dog is presented in Supplementary Figure 5D and Supplementary Figure 5G, respectively. The no observed adverse effect levels (NOAELs) of DTP3 in rat and dog were derived on the basis of the totality of the data from the 28-day repeat-dose toxicity studies conducted in these species (Figure 6C, Figure 6F).

PK Studies

In mouse, the PK parameters of DTP3 were abstracted from single-dose studies conducted at Cyprotex laboratories according to in-house SOP (http://www.cyprotex.com/admepk/bioanalysis/pharmacokinetics). DTP3 was administered to male mice of the NOD/SCID strain (NOD.CB17-*PrKdc*^{scid}/NcrCrl; Charles River) by intravenous or subcutaneous injection, as shown, over a dose range from 2.5 to 50 mg/kg (Supplementary Figure 3C-D). Three mice (20-25 g) were dosed per dose level, per time point. DTP3 was formulated in 1x PBS. For plasma preparation and drug analysis, blood samples were withdrawn 0.08, 0.25, 0.5, 1.0, 2.0, 4.0, 8.0, and 24 hours post-dosing, and quantified using an LC-MS/MS

methodology specifically developed to detect DTP3. Standard curves were prepared in blank plasma matrices and treated in an identical manner to the samples. The limit of quantification of the assay was 1 ng/mL. PK parameters were initially calculated at Cyprotex using non-compartmental analysis of the concentration profiles of DTP3 (Phoenix WinNonlin; Certara) (https://www.certara.com/software/pkpd-modeling-and-simulation/phoenix-winnonlin/?ap%5B0%5D=PKPD) (Supplementary Figure 3C-D).

The PK parameters of DTP3 in rat (Crl:WI Han; Charles River) and dog (Beagle; Harlan) were abstracted directly from the toxicokinetic studies performed at Covance laboratories in accordance with in-house SOP (https://www.covance.com/services/lead-optimization/pk-tk-analysis-and-reporting.html). Plasma samples were collected from male and female animals at various times after a 10-minute intravenous infusion of DTP3 at the indicated dose levels, on day 1 and day 28 of the 28-day repeat-dose toxicology studies (Supplementary Figure 3A-B), and quantified using an LC-MS/MS methodology specifically developed to detect DTP3. The lower limit of quantification of the assay was 1 ng/mL. PK parameters were initially calculated at Covance using non-compartmental analysis of the concentration profiles of DTP3 (Phoenix WinNonlin; Certara), up to 24 hours post-dosing on day 1, and up to 72 hours post-dosing on day 28 (Supplementary Figure 3A-B).

However, there was evidence of a significant distribution phase in the plasma profiles of DTP3 in both rodent and non-rodent species, and accounted for the majority of the area under the plasma concentration over time curve (AUC). There was also evidence of a longer underlying half-life, which was consistently underestimated using non-compartmental analysis. Consequently, the plasma concentration profiles of DTP3 were subsequently fitted to compartmental models in order to derive a more accurate assessment of terminal half-lives ($t_{½}$) (PK Solutions,

Summit Research Services; https://www.limswiki.org/index.php/Summit_Research_Services) (Figures 4A). Plasma clearance (CL) and volume of distribution (area) (V_a) were derived from the AUC and terminal half-life values. Accumulation ratios (RA) for systemic exposure parameters, *i.e.*, $AUC_{(0-t)}$ and C_{max}, were calculated by dividing the value on day 28 by the corresponding value on day 1 (Supplementary Figure 3A-B). Dose normalised values for C_{max} and $AUC_{(0-t)}$ were calculated by dividing the respective parameter by the nominal dose level (Supplementary Figure 3A-B). In the dose-ranging studies, AUC values were normalised for dose-level in order to investigate dose linearity.

For inter-species scaling purposes, estimates of the potential plasma half-life in humans were carried out using a conventional scaling methodology based on inter-species differences in liver blood flow, while assuming that systemic clearance would be the same proportion of liver blood flow in each species, and that volume of distribution would also be the same (4,5).

ADME Studies

The plasma protein binding (PPB) of DTP3, *in vitro*, was investigated in mouse (male; CD1 strain), rat (male; Sprague Dawley strain), dog (male; Beagle strain) and human (mixed sex) in two separate studies conducted on different batches of plasma at Cyprotex laboratories in accordance with in-house SOP (http://www.cyprotex.com/admepk/protein_binding/plasma-protein-binding) (Figures 4B). The metabolic stability of DTP3, *in vitro*, was also assessed in mouse, rat, dog, monkey and human hepatocytes at Cyprotex laboratories in accordance with in-house SOP (http://www.cyprotex.com/admepk/in-vitro-metabolism/hepatocyte-stability) (Supplementary Figure 3F). The tissue distribution, metabolism, and routes and rates excretion of DTP3 were investigated in rat at Covance laboratories using

liquid scintillation counting (LSC) and quantitative whole-body autoradiography (QWBA), according to in-house SOP (https://www.covance.com/services/analytical-services/drug-metabolism-and-pharmacokinetic-services.html). Briefly, male rats of the Han Wistar strain (Charles River) and a partially-pigmented male rat of the Lister-Hooded strain (Charles River) were dosed with 15 mg/kg of phenyl-U-[^{14}C]-tyrosine-labelled DTP3 (corresponding to a radioactivity of approximately 3.7 MBq/kg) by a 10-minute intravenous infusion via a lateral tail vein. A terminal blood sample was withdrawn by cardiac puncture from all animals and collected into tubes pre-coated with lithium heparin. Whole-blood aliquots were taken from each sample, and the residual blood sample was centrifuged to obtain plasma. Radioactivity concentrations in blood and plasma samples were measured using LSC (Supplementary Figure 3E). At pre-determined endpoints, 10 minutes and 1, 4, 24, 48, 72, and 168 hours post-dosing, the animals in the QWBA study were humanely killed by cold shock by plunging them into a freezing mixture, following deep anaesthesia under isoflurane. Once frozen, carcasses were prepared for analysis using in-house QWBA techniques (Figure 4C). The radioactivity in tissues was measured using a validated image analysis system (Supplementary Figure 3E).

After dosing, one animal from the QWBA study group (Lister-Hooded strain) and two additional animals (Han Wistar strain) were placed into glass metabolism cages for the collection of urine, faeces, and expired air for up to 168 hours post-dosing. Urine samples were collected over the following periods post-dosing: 0-7, 7-24, 24-48, 48-72, 72-96, 96-120, 120-144, and 144-168 hours. Faeces were collected over the following periods post-dosing: 0-24, 24-48, 48-72, 72-96, 96-120, 120-144 and 144-168 hours. At the end of the sampling period, the animal from the QWBA study group was subjected to the QWBA procedures, whilst the other two animals were humanely killed, and their carcasses digested in methanolic KOH. The radioactivity was determined in excreta and carcasses using LSC (Figure 4D).

Pooled plasma and faeces samples were prepared for metabolite profiling and identification by sequential extraction with acetonitrile. Pooled urine samples were centrifuged to remove particulate material. For each sample, metabolite profiles were generated using radio-HPLC. On-line radio-detection was used for urine and faeces, whereas off-line radio-detection was used for plasma, due to the low radioactivity levels present in plasma samples. Selected samples were further analysed by high resolution LC-MS(/MS) in order to characterise the most notable metabolites in each matrix (Figure 4E, Supplementary Figure 3G).

Statistical Analyses

Results were expressed as the mean of the indicated number of samples. The statistical error was calculated as either standard deviation (SD) or standard error of the mean (SEM), as indicated, according to the typology of the experiments. Statistical analyses of the results from the studies conducted at external contract research organisations (CRO) were conducted in accordance with in-house SOP. Statistical analyses of the results from in-house experiments (Figure 5A and Figure 5C) were conducted using two-tailed t test. In all cases, a value of $p<0.05$ was considered to be statistically significant.

SUPPLEMENTARY REFERENCES

1. Tornatore L, Sandomenico A, Raimondo D, Low C, Rocci A, Capece D, *et al*. Cancer-selective targeting of the NF-κB survival pathway with GADD45β/MKK7 inhibitors. *Cancer Cell* **2014**; 26: 495-508.
2. Riccardi C, Nicoletti I. Analysis of apoptosis by propidium iodide staining and flow cytometry. *Nat. Protoc.* **2006**; 1: 1458-1461.

3. Irwin S. Comprehensive observational assessment: Ia. A systematic, quantitative procedure for assessing the behavioral and physiologic state of the mouse. *Psychopharmacologia* **1968**; 13(3): 222-257.
4. Ward KW, Smith BR. A comprehensive quantitative and qualitative evaluation of extrapolation of intravenous pharmacokinetic parameters from rat, dog, and monkey to humans. I. Clearance. *Drug Metab. Dispos.* **2004**; 32: 603-611.
5. Evans CA, Jolivette LJ, Nagilla R, Ward KW. Extrapolation of preclinical pharmacokinetics and molecular feature analysis of “discovery-like” molecules to predict human pharmacokinetics. *Drug Metab. Dispos.* **2006**; 34: 1255-1265.

SUPPLEMENTARY FIGURE LEGENDS

Supplementary Figure 1 – Related to Figure 1.

The secondary pharmacology and drug-drug interaction potential of DTP3. **A**, List of the 80 validated drug targets tested in the radio-ligand competition binding assays shown in Figure 1A. **B**, Radio-ligand competition binding assays showing the dose-dependent inhibition by DTP3 of the binding of Sigma receptors (non-selective) to the agonistic ligand, haloperidol. Values denote the means of the percentage of haloperidol binding in the presence of the indicated DTP3 concentrations (n=2). **C**, Table reporting the IC_{50} and K_i values of DTP3 for Sigma receptors (non-selective) calculated from the assay shown in (**B**). Also reported are the historic IC_{50} and K_i values of haloperidol for Sigma receptors for assays conducted at CEREP. **D**, Functional assays showing the agonistic (top) and antagonistic (bottom) effects of DTP3 at the indicated concentrations on Sigma receptors (non-selective). Also reported are the dose-dependent effects on Sigma receptors of the control agonist, (+)SKF-10,047, and the control antagonist, rimcazole. Values represent the mean percentage of variation of the response to the reference Sigma-receptor ligand in the

presence of DTP3 or control agonist or antagonist relative to the response measured in the absence of any other agents. **E**, Heat map reporting the time-dependent inhibition of the indicated CYP isoforms by DTP3 at the indicated concentrations. Shown are the percentages of isoform-specific CYP activity measured in the presence (30 min) or absence (0 min) of a 30-minute pre-incubation of human liver microsomes with or without NADPH, as indicated. **F**, Time-dependent inhibition assays showing the inhibition of the indicated CYP isoforms by specific control compounds in the presence or absence of a 30-minute pre-incubation of human liver microsomes with or without NADPH, as indicated. IC_{50} values denote the concentrations of control compounds resulting in a 50% inhibition of the formation of isoform-specific metabolites in each condition shown.

Supplementary Figure 2 – Related to Figure 2.

Absence of off-target hepatocellular cytotoxicity and genotoxicity by DTP3. **A**, qRT-PCR showing the relative *GADD45B* mRNA expression levels in the human hepatocellular carcinoma cell line, HepG2, and the indicated positive and negative control cell lines, U266 and HEK-293T, respectively. Values denote means ± SD (n=3). **B**, Bacterial reverse mutation assays (Ames test) showing the fold increase in colony number for the indicated histidine-requiring reporter strains of *Salmonella typhimurium* (*i.e.*, TA98, TA100, TA1535, TA1537, and TA102) following exposure to DTP3 at the concentrations of 5, 16, 50, 160, 500, 1,600, and 5,000 µg/plate or the strain-specific positive control compounds (CTR) shown in (**C**), in the presence (bottom) or absence (top) of metabolic activation by Aroclor 1254-induced rat liver post-mitochondrial S9 fraction. Values denote the mean fold increase in colony numbers observed in the presence of DTP3 or control compounds (n=3) relative to the colony number observed with vehicle control (n=5). **C**, List of the positive control compounds (CTR) used in (**B**) and their application in the assay.

Supplementary Figure 3 – Related to Figure 4.

The PK and ADME profiles of DTP3. **A**, Table reporting the values of the indicated toxicokinetic parameters of DTP3 in rat on day 1 (top) and day 28 (bottom) following administration by rapid intravenous infusion at the indicated dose levels during the 28-day repeat-dose toxicity study. **B**, Table reporting the values of the indicated toxicokinetic parameters of DTP3 in dog on day 1 (top) and day 28 (bottom) following administration by rapid intravenous infusion at the indicated dose levels during the 28-day repeat-dose toxicity study. **C**, Table reporting the values of the indicated PK parameters of DTP3 in mouse following administration by intravenous injection at the indicated dose levels. **D**, Table reporting the values of the indicated PK parameters of DTP3 in mouse following administration by subcutaneous injection at the indicated dose levels. **E**, Quantitative whole-body autoradiography (QWBA) and liquid scintillation counting (LSC) reporting the concentrations of DTP3-related radioactivity in the indicated tissues of male rats at the times shown following rapid intravenous infusion of 15 mg/kg of [^{14}C]-labelled DTP3. Radioactivity was quantified by LSC in blood and plasma samples and by QWBA in the other tissues. Representative images of the QWBA analysis are shown in Figure 4C. **-**, tissue concentration below the lower limit of quantification. **F**, LC-MS(/MS) analysis showing the metabolic stability of DTP3 (3 µM) and the control compounds, verapamil (VRP) and umbelliferone (UBF), following a 120-minute (DTP3) or 60-minute (VRP and UBF) incubation with primary hepatocytes from rat, dog, monkey and human, as indicated. Values denote the mean intrinsic clearance (CL_{int}) of each compound. DTP3, n=6; VRP, 4≤n≤6; UBF, 3≤n≤5. The measured CL_{int} values characterise DTP3 as a low clearance compound in all species investigated, according to the following assay classification thresholds: human, <3.5; rat, <5.1; dog, <1.9; monkey, <5.2. **G**, LC-MS(/MS) analysis showing the quantification of radio-labelled DTP3 and the

indicated radioactive DTP3 metabolites in plasma from rats at the times shown following administration of [^{14}C]-DTP3 as in (**E**). **A-D**, C_{max}, maximum plasma concentration; C_{max}/D, dose-normalised C_{max}; $AUC_{(0\text{-}t)}$, area under the plasma concentration over time curve from time 0 to the last measurable concentration; $AUC_{(0\text{-}t)}$/D, dose-normalised $AUC_{(0\text{-}t)}$. **A**, **B**, $AUC_{(0\text{-}\infty)}$, area under the plasma concentration over time curve from time 0 to infinity; RA_{Cmax}, accumulation ratio based on C_{max}; RA_{AUC}, accumulation ratio based on $AUC_{(0\text{-}t)}$; M, male; F, female. **C**, **D**, $AUC_{(0\text{-}24)}$/D, dose-normalised $AUC_{(0\text{-}24)}$.

Supplementary Figure 4 – Related to Figure 5.

The therapeutic efficacy of DTP3 in a mouse MM xenograft model upon intravenous bolus injection by a clinically suitable dosing schedule. **A**, Histograms showing the FACS analysis of phosphorylated (phospho-)JNK (top), cleaved caspase 3 (middle) and phospho-ERK (bottom) in $CD138^+$ U266 cells isolated from subcutaneous MM xenografts in representative mice treated with vehicle (CTR) or 10 mg/kg of DTP3 on days 1, 3, 5, and 8, according to the clinical dosing schedule, and analysed approximately 24 hours after the last dosing on day 8. Depicted are the signals resulting from the intracellular staining with phospho-JNK-specific, phospho-ERK-specific, or cleaved caspase 3-specific antibodies (blue) or appropriate immunoglobulin isotype controls (red). **B**, Dot plots (top) and nucleosomal DNA fragmentation assays (bottom) showing the FACS analysis of the cellular profile and apoptotic cells, respectively, in representative tumours from (**A**). The percentages of apoptotic cells (bottom), containing a sub-G_1 DNA content, are depicted.

Supplementary Figure 5 – Related to Figure 6.

The toxicological profile of DTP3. **A**, Experimental design for the 28-day repeat-dose toxicity study of DTP3 in rat. Shown are the numbers and sexes of the animals for

each experimental group in the main toxicity study, the toxicokinetics study, and the delayed-onset toxicity and reversibility of toxicity study. **B**, Table reporting the list of in-life procedures performed in (**A**) and the corresponding 28-day repeat-dose toxicity study in dog (**E**), including clinical observations and clinical laboratory procedures. *, procedure performed exclusively in dog; **, parameter evaluated semi-quantitatively; #, inclusive of platelet clump assessment. **C**, Table reporting the list of clinical chemistry and urinalysis parameters of kidney function in rats from (**A**). Values denote means ± SD or the number of animals within each range of measurements, as shown. The number of animals in each group (n) is depicted. **D**, Heat-map showing the percentage of rats exhibiting drug-related post-dosing macroscopic and/or microscopic abnormalities in the listed organs and tissues at the terminal kill following treatment with vehicle control (CTR) or the indicated doses of DTP3 as in (**A**). **E**, Images of H&E staining showing the kidney histology in either representative rats from (**A**) or rats with lesions of worst severity (grade 2) in the groups treated with 100 mg/kg of DTP3 at the terminal kill (**A**), as indicated. Arrows denote a typical kidney tubular basophilia. The scale is shown. **F**, Experimental design for the 28-day repeat-dose toxicity study of DTP3 in dog. Shown are the numbers and sexes of the animals for each experimental group in the main toxicity study (including the toxicokinetics study) and the delayed-onset toxicity and reversibility of toxicity study. **G**, Heat-map showing the percentage of dogs exhibiting drug-related post-dosing macroscopic and/or microscopic abnormalities in the listed organs and tissues at the terminal kill following treatment with vehicle control (CTR) or the indicated doses of DTP3 as in (**F**). **A**, **C-G**, M, male; F, female. **C**, **G**, *, tissue not processed or not evaluated microscopically.
